# The Tiny *Drosophila Melanogaster* for the Biggest Answers in Huntington’s Disease

**DOI:** 10.3390/ijms19082398

**Published:** 2018-08-14

**Authors:** Abraham Rosas-Arellano, Argel Estrada-Mondragón, Ricardo Piña, Carola A. Mantellero, Maite A. Castro

**Affiliations:** 1 Unidad de Imagenología, Instituto de Fisiología Celular, Universidad Nacional Autónoma de México, Ciudad de México 04510, Mexico; 2 Department of Clinical and Experimental Medicine, Linköping University, 581 83 Linköping, Sweden; argel.estrada@comunidad.unam.mx; 3 Laboratorio de Neurociencias, Departamento de Biología, Facultad de Química y Biología, Universidad de Santiago de Chile, Santiago 9160000, Chile; ricardop@ug.uchile.cl; 4 Departamento de Ciencias Químicas y Biológicas, Universidad Bernardo O’Higgins, Santiago 8370993, Chile; 5 Facultad de Ciencias de la Salud, Universidad de Las Américas, Santiago 7500972, Chile; carola.mantellero@usach.cl; 6 Instituto de Bioquímica y Microbiología, Facultad de Ciencias, Universidad Austral de Chile, Valdivia 5090000, Chile; 7 Center for Interdisciplinary Studies on the Nervous System (CISNe), Universidad Austral de Chile, Valdivia 5090000, Chile

**Keywords:** neurodegenerative diseases, IT15, LOMARS, HTT, HD, huntingtin, mHTT, polyQ, polyglutamine disorders, chorea, neostriatum, fruit fly

## Abstract

The average life expectancy for humans has increased over the last years. However, the quality of the later stages of life is low and is considered a public health issue of global importance. Late adulthood and the transition into the later stage of life occasionally leads to neurodegenerative diseases that selectively affect different types of neurons and brain regions, producing motor dysfunctions, cognitive impairment, and psychiatric disorders that are progressive, irreversible, without remission periods, and incurable. Huntington’s disease (HD) is a common neurodegenerative disorder. In the 25 years since the mutation of the huntingtin (*HTT*) gene was identified as the molecule responsible for this neural disorder, a variety of animal models, including the fruit fly, have been used to study the disease. Here, we review recent research that used *Drosophila* as an experimental tool for improving knowledge about the molecular and cellular mechanisms underpinning HD.

## 1. Introduction

Living longer does not necessarily imply a healthy life. The increase in the elderly population is considered a human health phenomenon of global importance [1,2]. The average life expectancy, or the statistical measure of how long a person is likely to live based on demographic factors, has increased over the years. According to the World Health Organization (WHO), mankind has globally gained an additional 41 years of life over the past 200 years. It is estimated that individuals born in 2016 will live on average 72 years; 74.2 if female or 69.8 if male (available at www.who.int/gho/mortality_burden_disease/life_tables/situation_trends_text/en/, accessed on 30 July 2018). However, the extended lifespan and the quality of life do not correlate. In 2015, the WHO estimated that a healthy life expectancy is up to 65 years, although not in all cases. At the end of adulthood (60–65 years) and to the elderly (66+ years), non-genetic (demographic and environmental) features and genetic vulnerability (dominant associated factor) are undisputed risk factors for the appearance of neurodegenerative diseases with sporadic global incidence. Age-dependent disorders have become a worldwide challenge and are receiving global attention because they are progressive, uninterrupted, irreversible, incurable, and unfortunately, lack effective treatment. Some of the most common neurodegenerative disorders are Alzheimer’s, Parkinson’s, Amyotropic Lateral Sclerosis, and HD [3].

Despite the fact that aging is a common shared feature of several neurodegenerative diseases, HD is a disorder with a variable age onset, characterized by a comparatively early age of disorder onset, resulting in a juvenile form of HD [3], detailed below. However, as in the other neurodegenerative diseases, the HD genetic factors are strongly related with risk factors, but only weakly associated with environmental or demographic factors [3].

A wide variety of animal models have been designed for the study of human neurodegenerative diseases, and they play a crucial role in studying the molecular, physiological, and behavioral principles that underlie each pathology. A high similarity between the human pathology and animal model phenotypes is an advantage, because the latter do not typically acquire the same genetic disease in a natural way. Twenty-five years ago, the abnormal polyglutamine repeat motif was identified as the *HTT* gene mutation responsible for HD [3]. Since then, several vertebrate and invertebrate models have allowed us to experimentally reproduce the genetic and molecular mechanisms that are characteristic of the human disease. Currently, genetic animal models are the most valuable tools that provide trustworthy information in order to understand the systemic and molecular dynamics, develop novel therapeutic strategies, and improve the quality of life of the affected patients and their offspring. Genetic HD animal models include transgenic, knock-in, and gene mutations in nonhuman primates, sheep, pig, mouse, zebrafish, and the fruit fly [4,5,6,7,8,9,10]; as well as diverse cell line models such as HdhQ7/111 and STHdhQ111 [11,12,13].

This review provides an update about the role and legacy of the fruit fly as an HD animal model without a polyglutamine (polyQ) motif in the corresponding homologous region of the N-terminal domain of its wild-type htt protein; as well as diverse genetically designed *Drosophila* models for the study of HD.

## 2. Neurodegenerative Diseases

As was mentioned above the majority of neurodegenerative diseases are produced by specific combinations of genetic predispositions, age onset, and environmental factors. Their effects are characterized by proteostasis dysfunction in vulnerable cells of different anatomical structures within the nervous system [14]. These disorders typically have a late onset and slow progression over time, with aging being an important factor of gradual cell damage build-up/death. Furthermore, such specific damage takes place in a defined population of neurons, based on the susceptibility of each disorder (i.e., dopaminergic *substantia nigra pars compacta* neurons for Parkinson’s disease, projection neurons of the hippocampus and enthorhinal cortex for Alzheimer’s disease, spinal motoneurons for Amyotropic lateral sclerosis, and striatal medium-sized spiny neurons (MSSNs) for HD [14]). Depending on the identity of the disease and its selective neuronal vulnerability in different stages, this will produce psychiatric conditions, cognitive disabilities, general motor impairment, dementia, and finally, death, all of which are hallmarks of neurodegenerative diseases [14,15].

Interestingly, inherent protein aggregates in a particular neurodegenerative disease are the result of the mutated version of otherwise ubiquitously expressed genes. Despite the fact that each neurodegenerative disease can affect a particular brain region, neural networks, or a specific subpopulation of neural cells; the corresponding subcellular targets that are damaged, such as mitochondria and endoplasmic reticulum (ER); and the related intracellular processes, including autophagy, proteostasis, and ER stress, are common features in several neurodegenerative disorders like Alzheimer’s, Parkinson’s, and HD [15]. These three pathologies have their selective mutant protein aggregates, namely: Beta-amyloid and tau for Alzheimer’s disease, alpha-synuclein for Parkinson’s disease, and mutant HTT for HD [3,15,16,17].

## 3. Wild-Type and Mutant Huntingtin

**Figure 1:**
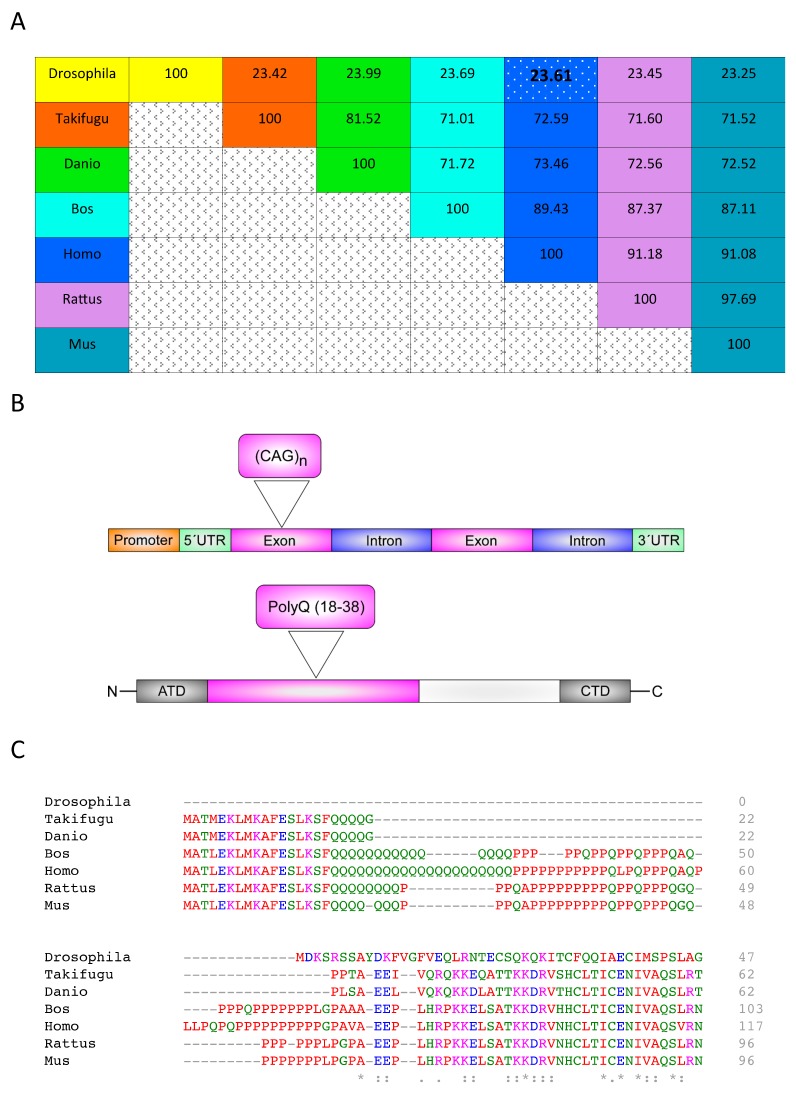
Multiple sequence analysis of huntingtin protein using Clustal Omega. (**A**) Percent identity matrix of the HTT proteins of several species (*Takifugu rubipres* in orange; *Danio rerio* in green; *Bos taurus* in cyan; *Rattus norvegicus* in purple; *Mus musculus* in blue aqua color); the homologies between *Homo sapiens* (blue) and *Drosophila melanogaster* (yellow) are highlihted. (**B**) Linearized *HTT* gene and protein showing the CAG location and polyQ region. (**C**) Multiple sequence analysis of polyQ and polyP repeats of HTT protein. All figures using protein sequences of mammalian (*Homo sapiens*, UniProtKB—P42858; *Mus musculus*, UniProtKB—P42859; *Bos taurus*, UniProtKB—E1B8E7; and *Rattus norvegicus*, UniProtKB—G3V9P7), fish (*Danio rerio*, UniProtKB—O42269, and *Takifugu rubripes* UniProtKB—P51112), and fruit fly (*Drosophila melanogaster*, UniProtKB—Q9V3N4) species. An asterisk (*) indicates positions which have a single, fully conserved residue. A colon (**:**) indicates conservation between groups of strongly similar properties. A period (**.**) indicates conservation between groups of weakly similar properties.

The huntingtin gene, abbreviated as *HTT* in *Homo sapiens* (Human Genome Organization [HUGO], Gene Nomenclature Committee), is also known as IT15 (interesting transcript 15), HD, and LOMARS (Lopes–Maciel–Rodan Syndrome). *HTT* is a gene with a large locus, spanning 180 kb and consisting of 67 exons. It is expressed as two alternatively polyadenylated forms; the first is a larger transcript (13.7 kb) than the second (10.3 kb). Throughout life, the larger *HTT* transcript is constitutively expressed in the nervous system, whereas the smaller transcript is expressed in other organs (NCBI, Gene ID: 3064). *HTT* is translated into a multifunctional protein also called HTT, which is required from early development to later stages of life. HTT is involved in vesicular transport, endocytosis, autophagy, and transcriptional regulation [18,19,20]. HTT, alternatively called Huntington’s disease protein or, simply, HD protein (UniProtKB—ID: P42858), in vertebrates, is a highly conserved soluble 348 kDa protein [21] (Figure 1A). There are few similarities in the sequence between humans and other species, which also occurs with the orthologous gene of the fruit fly. The *HTT* gene is located in (data are provided with the official abbreviation of each species) chromosome 1 in zebra fish (*htt*, ID: 30214), 4 in human (*HTT*, ID: 3064), 5 in mouse (*Htt*, ID: 15194), 6 in cattle (*HTT*, ID: 615059), 14 in rat (*Htt*, ID: 29424), 17 in Japanese pufferfish (*htt*, ID: 101065600), and 3R in fruit fly (*htt*, ID: 43392). A full analysis of the primary protein sequence alignment of these representative species is illustrated in Supplementary Figure S1. The HTT protein is related to several cellular functions, such as embryonic development (lethal during the development of knockout mice), intracellular transport of molecules (such as vesicle trafficking), apoptosis (prevents caspase activation), and transcription regulation (in brain-derived neurotrophic factor expression) [22,23,24,25].

In humans, an important and distinctive characteristic of HTT consists of a short and repeated sequence of glutamines, known as the polyQ motif, located at the N-terminal domain. The polyQ domain is encoded by a cytosine-adenine-guanine (CAG) triplet repeat in the first exon of the gene (Figure 1B). The total polyQ length is longest in humans and is not conserved amongst different species (Figure 1C). Interestingly, the number of glutamines decreases between the lineages that were separated early in evolution [21]. The polyQ sequence appeared in the HTT protein for the first time in the evolutionary timeline in fish. Its physiological function is related to the recognition mechanism of transcription factors and other protein-protein interactions [23].

Altered expandable repeats of the CAG trinucleotide encode for glutamine, resulting in abnormal polyQ repeats, also known as the polyglutamine tract. These abnormal polyQ repeats are the hallmark of incurable, hereditary, and degenerative disorders called polyQ diseases, which include spinal and bulbar muscular atrophy (35 - 38 CAG repeat size), dentatorubropallidoluysian atrophy (49 - 88 CAG repeat size), and various spinocerebellar ataxias (SCA1, 39 - 82 CAG repeat; SCA2, 34 - 64 CAG repeat; SCA3, 60 - 84 CAG repeat; SCA7, CAG repeat 34 - 306) [26,27,28]. Unaffected people have a range between 6 - 35 CAG repeats, with 99% of such individuals having <30 repeats; however, those with HD have 36 - 121 CAG repeats [29,30,31]. Expandable CAG repeats can increase via inheritance, raising the probability of longer triplet repeats and early HD onset in successive generations. It is also assumed that the repeat expansion mechanism occurs during DNA replication. The following factors have been suggested: (1) DNA strand slippage during replication; (2) looping of one or several repeats in the newly synthesized DNA strand; and (3) unusual structural characteristics, such as hairpin-like structures that give rise to slip-outs that are folded into these DNA structures, being the Okazaki initiation zone, a region that could facilitate the unusual secondary structure [32]. Accumulation of defective HTT proteins induces its stacking in the nucleus, producing internal anomalous functions in addition to mitochondrial dysfunction and impairments commonly found in the proteasome system, in axonal transport, and in calcium homeostasis [33,34,35].

## 4. Huntington’s Disease

In the mid-19th century, George Huntington described this neurodegenerative disorder for the first time [36] as a hereditary, progressive illness characterized by clonic spasms and a lack of remission periods. Initially, HD was known as Huntington’s chorea, St. Vitus’ dance (a derogatory term), chorea of the aged, choreic dementia, dementia choreica, Osler’s preference, and chronic progressive chorea. It was not until the late 1960s that it received the name of HD [37].

HD is considered an infrequent neurological disorder with devastating consequences on quality of life. It affects women and men alike and the onset is between the ages of 30 and 55 years old. Moreover, a juvenile form of HD affects children and teenagers (cases with CAG repeats of 55 or greater, instead of over 40 in adult-onset HD). The prevalence and incidence rate of HD worldwide is unclear. A poor case ascertainment and inadequate diagnosis contribute to this important gap in our knowledge. Data published in 2016 by Rawlins and colleagues [38] suggested a prevalence in principal regions in the world as follows: (data are an average prevalence per 100,000 [95% confidence indexes {CIs}] and trend [percent] by decade, respectively) Asia 0.40 (0.36–0.44), 8.9 (–2.24 to +23.8); Central and Eastern Europe 2.17 (1.95–2.41), 15.4 (2.70 to +38.6); North America 7.33 (6.94–7.74), 20.1 (+18.1 to 22.1); Oceania 5.63 (5.61–6.25), 15.4 (+11.6 to +19.3); United Kingdom 6.68 (6.40–6.97), 15.5 (+11.3 to +18.0); and Western Europe 3.60 (3.50–3.69), 16.5 (+14.9 to +18.6).

As mentioned above, HD is an adult-onset, autosomal dominant polyQ disease that produces three major imbalances, namely: (A) Motor dysfunction, such as muscle spasms, uncontrollable jerking movements, rigidity, and speech problems. (B) Cognitive deficits, namely learning difficulties, difficulties in areas of planning and prioritizing, and impairments in spatial perception. (C) Psychiatric comorbid disorders including depression, personality changes, anxiety, delirium, mania, and dementia. In sum, these imbalances constitute the pathognomonic features of HD [39,40]. Another emerging distinguishing characteristic of HD is a peculiar signal at the cytological level. Mutant *HTT* carries a dominant toxic property that results in major cell damage and death in the basal ganglia. Specifically, this toxicity causes large neostriatal atrophy in a distinctive population of neostriatal projection neurons, known as MSSNs, expressing dopamine D2 receptors/encephalin-containing neurons [41,42,43,44,45,46,47]. Correspondingly, HD induces clear alterations at the neurochemical and receptor levels in the neostriatum [45,48,49,50,51,52,53,54,55].

## 5. Neostriatum and Central Complex as Homologous Neuroanatomical Structures

The anatomical nomenclature of the neostriatum is given to the collective conjunction of the caudate and putamen structures of the brain, sometimes called corpus striatum or, simply, striatum; however, the term striatum is also used to refer to the anatomical complex formed by the caudate and lentiform nucleus (formed by the putamen and *globus pallidus*). In accordance with the former nomenclature, we used the neuroanatomical term neostriatum in this review to refer to the caudate and putamen, as it has been used previously [56,57]. The neostriatum consists of three main regions, dorsal, ventral, and amygdaline. All regions are composed of matrix and striosomes (also known as patches) [58]. The neostriatum is the first to relay information between the cerebral cortex input and basal ganglia output, and it has been associated with motor control, learning, memory, and the emotional part of movement (amygdaline region) [59,60,61]. Neostriatum cytology is formed by two general cell groups, spiny or projection neurons, and aspiny or interneurons. The former group is also known as medium spiny neurons or MSSNs. This kind of neurons has cellular bodies between 10 and 20 μm in diameter, and dendritic trees with abundant spines and a dendritic extension of up to 500 μm in diameter. All MSSNs are gamma-aminobutyric acid (GABA) -containing neurons and represent the most abundant neostriatal neuron type, making up 95% of the total neuronal population [56]. MSSNs synthetize neuropeptides differentially in two selective populations, one corresponds to substance P projection neurons, in addition to the dopamine D1 receptor (called the direct pathway), and the other corresponds to enkephalin projection neurons, which express the dopamine D2 receptor (called the indirect pathway) [62,63]. Functionally, the enkephalin/D2 neostriatal inhibitory neurons have opposite effects (compared to substance P/D1 excitatory neurons) on the neural activity of their downstream synaptic targets [64] and are involved in the facilitation of planning movements of the trunk and limbs, and in the inhibition of involuntary movements [64,65].

Vertebrate basal ganglia and the arthropod central complex derive from embryonic basal forebrain lineages that are specified by an evolutionary conserved genetic program leading to interconnected neuropils and nuclei that populate the midline of the forebrain–midbrain boundary region; in addition, gene expression patterns are specific to the region of the developing brain, corresponding to that of the vertebrates. The *Dlx1/2*, *Tlx*, *Nkx2.1*, *Pax6*, *Emx2*, *Gsh1/2*, and *Lhx6/7* genes play essential roles in the development and specification of the neostriatum, and have homologs in *Drosophila FGF8*, *SHH*, *BMP*, *Otx2*, *Dlx1/2*, *Tlx*, *Nkx2.1*, *Pax6*, *Emx2*, *Gsh1/2*, *En1/2*, and *Pitx2* [66]. Moreover, the region-specific gene expression patterns in the developing brain correspond to those in vertebrates. Therefore, the central complex of arthropods and the vertebrate basal ganglia are homologous brain structures that both share topography and embryological derivation, as well as functionality and gene expression [66,67]. The central complex structure is highly conserved across the insect species [68]. Functionally, the central complex of *Drosophila* is involved in start–stop and turning locomotor activity, walking activity, speed, leg coordination, restlessness, flight behavior, control and fine tuning of behavior [69,70,71]. This neuroanatomical structure is strongly related to the integration of data from the two brain hemispheres, consisting of the following four interconnecting midline neuropils: protocerebral bridge, fan-shape body, ellipsoid body, and paired noduli, where at least 15 neuronal lineages have been identified [71,72,73]. The majority of these neuron classes have been categorized as large-field and small-field neurons. The former group typically arborize within one or more tangential layers of the central complex, and they form networks with one or more accessory structures outside of the central complex; whereas the latter group interconnect neuropils of the central complex and a subpopulation project into other brain regions [68]. Interconnections between neuropils are restricted; fan-shape body neurons connect with specific regions of noduli, while the protocerebral bridge make its particular connection with the noduli through the ellipsoid body [74]. Structural hallmarks of the neostriatum include the neuronal arrangement in the striosomes and matrisomes, whereas in the fly central complex, neuropils display several arrangements, namely: the protocerebral bridge is divided into sectors or segments, the fan-shaped body compromises of an arrangement of successive synaptic layers intersected by repeated arrangement modules, the ellipsoid body is subdivided into three axes called rings, whereas noduli have several subdomains named dorsal, medial, and ventral compartments, suggesting a high degree of regional specialization into the central complex [74]. In vertebrates, the D1 pathway has a net positive effect on the basal ganglia output, while the D2 pathway has a negative effect. In *Drosophila*, clusters of dopamine-containing D1 neurons have specific projection patterns, and some clusters are associated with mushroom bodies, the central complex, fan-shaped body, ellipsoid body, and lateral accessory lobes. Of course, all of these similarities do not exclude the possibility that some of these pathways may retain parallel functions or compensatory co-adapted circuits. The multiple commonalities mentioned here only demonstrate the deep homology of the arthropod central complex and vertebrate basal ganglia. Functionally the central nervous system (CNS) specific depletion of dopamine in *Drosophila* results in reduced activity, locomotor deficits, extended sleep time, and defects in the aversive olfactory memory formation, suggesting that arousal and choice require normal dopamine levels. The perturbation of dopaminergic pathway activity, or its modulatory output interferes with the corresponding behavioral actions, common in age-related degeneration of the dopaminergic clusters, and typical of HD. As in the neostriatum, the central complex displays a strong GABA neurotransmitter and receptor expression, at least in the ellipsoid body neuropil [66,67].

## 6. The *Drosophila* Huntingtin

**Figure 2:**
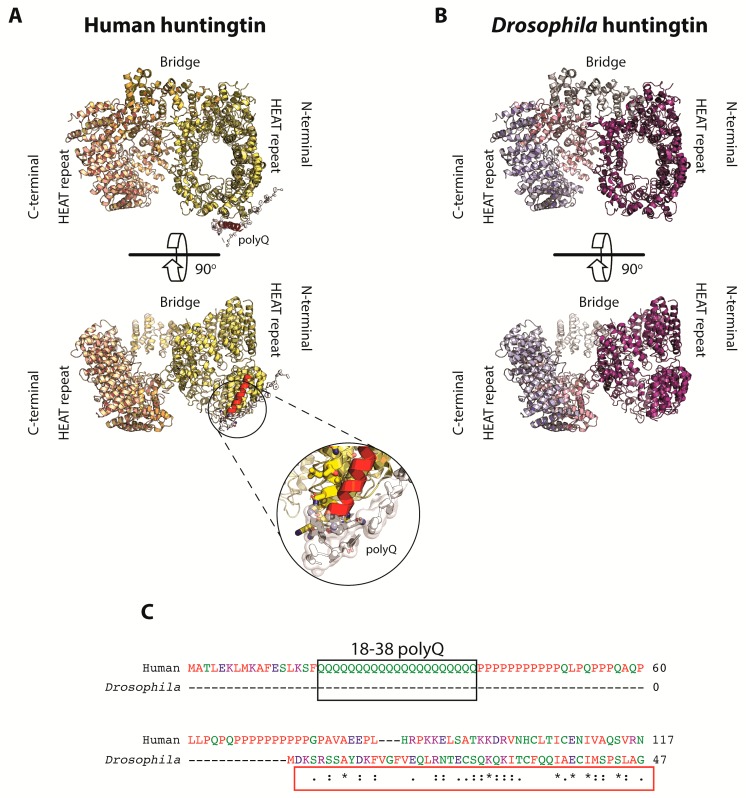
Comparison between *Homo sapiens* and *Drosophila melanogaster* huntingtin proteins. (**A**) Architecture of *Homo sapiens* HTT with its different domains in two different planes, with a rotation of 90 degrees on the X-axis. The N-terminal domain begins with an α-helix continued for a stretch of polyglutamines/polyprolines not present in the homologous version of this protein in *Drosophila melanogaster*. Such a domain is zoomed in to highlight its key role and interaction with the rest of the protein for the development of HD. HEAT repeats domains are represented by C-terminal (ochre), bridge (yellow) and N-terminal (pale yellow). (**B**) Architecture of *Drosophila melanogaster* htt showing its different domains in two different planes, represented in the same way as the *Homo sapiens* homologous version. HEAT repeats domains are represented by C-terminal (purple blue), bridge (purple violet) and N-terminal (purple deep). (**C**) Alignment of the first part of the N-terminal domain of the *Homo sapiens* and *Drosophila melanogaster* huntingtin protein sequences, identifying the polyglutamine stretch as relevant for HD development in the *Homo sapiens* sequence but not in the *Drosophila melanogaster* sequence. The figure was created using the Protein Data Bank entries 3io4 and 6ez8 [20,81]. An asterisk (*) indicates positions which have a single, fully conserved residue. A colon (**:**) indicates conservation between groups of strongly similar properties. A period (**.**) indicates conservation between groups of weakly similar properties.

Unlike humans, *Drosophila* htt does not express an expanded polyQ sequence in its amino terminal domain. Nevertheless, *Drosophila* and human proteins share around of 49% positive homology and 27% identity. Therefore, the considerable sequence conservation between the two species predicts a common folding for both proteins. Human HTT is a large 348 kDa protein and was recently studied by cryo-electron microscopy at an overall resolution of 4 Å. It consists of three domains, two of which are the N-terminal and C-terminal domains containing multiple tandem repeats of a structural motif composed of two alpha helices linked by a short loop [20]. The tandem sequence was identified in the following four archetypical proteins: the representative **H**untingtin, elongation factor 3 (**E**F3), protein phosphatase 2A (PP2**A**), and yeast kinase **T**OR1, collectively known as HEAT [75]. The HEAT repeats are linked by a third and smaller bridge domain. The N-terminal HEAT repeat forms an α-solenoid, comprising of 21 HEAT repeats arranged as a one and a half-turn right-handed superhelix. The C-terminal HEAT repeat comprises of 12 HEAT repeats forming an elliptical ring. Many recent structural studies have focused on the N-terminal fragment corresponding to the first exon of the *HTT* gene, where the polyQ tract is located, and which remains largely unknown [20]. Figure 2 contains a model that represents a possible mechanism of how this domain interacts with the rest of the N-terminal huntingtin protein in humans, not in *Drosophila*. Interestingly, the htt protein sequence is highly conserved between the *Drosophila* species, suggesting a common biological function in the fruit fly [76,77], and a key tool to study the main functions of this protein in this animal model. Similar to vertebrates, htt protein is transported bi-directionally in *Drosophila’s* axons, where it is associated with several proteins that receive the name of huntingtin associate proteins (HAP), with HAP40 being a key regulator of endocytosis, a critical factor for HD development in humans [78]. No HAP40 homologue appears to be present in *Drosophila*, suggesting that these two proteins may have co-evolved in vertebrates, and their interactions could be important for the appearance of HD in humans [20]. In any case, some other huntingtin interacting proteins are dynactin, dynein intermediate chain, and kinesin 1 [22,79,80], which are still under extensive research.

## 7. The Legacy of Fly Models in HD

### 7.1. Drosophila melanogaster as an Animal Model

*Drosophila* is a well-known model organism that plays a critical role in biological sciences; moreover, the fruit fly has been recognized as an indispensable tool in neurobiological, biomedical, and developmental biology research [82]. Despite apparent differences between these organisms and vertebrates, several molecular mechanisms are highly conserved across evolution, such as the body axes pattern, wiring of a complex nervous system, organogenesis, and control of cell proliferation [83,84]. Exceptionally, many biological processes are common between fruit flies and humans, and these intrinsic similarities have allowed us to elucidate crucial mechanisms such as inheritance patterns [85], genetic mutations [86], genetic control of development [87], innate immunity control [88], and biological rhythms [89]. These and other research findings highlight *Drosophila* as an indispensable animal model.

Humans and fruit flies displayed an evolutionary divergence of about 783 million years. A total of 14.9% of human genes and 46% of *Drosophila* genes have orthologs to one or more fly and human genes, respectively [90]. Powerful genetic techniques have been used in *Drosophila* research and have proven useful for producing mutant models for the study of molecular processes (i.e., for biometal kinetics and mechanisms using chimeric proteins) [46,91,92], and to analyze human diseases; over five hundred *Drosophila* genes have been clearly related to human disease genes, including polyQ disorders [26,84]. The fruit fly is considered an exceptional model system to study neurodegenerative diseases [93]. Transgenic fruit flies can reproduce several characteristics of these disorders, including reduced longevity; late onset; motor syndromes; gradual accumulation of aggregates in neurons, axons, and cytoplasm; and consequently, neurodegeneration itself. In HD, *Drosophila* has been used as an animal model to report whether the distinct pathogenic elements of polyQ disorders are due to altered protein behavior, or to an inherent cytotoxic influence by enlarged polyQ chains [79,93].

In many of the cases referred to herein, the *Drosophila* model system has shown a plethora of molecular aspects that have enlightened us with fundamental elements to better understand the appearance and progression of the disease. Many of these studies performed on the *Drosophila* model can be directly extrapolated to the development of human HD, and they promise to be a valuable source of knowledge to palliate the disease by assisting in the development of new and effective drugs. These drugs should be able to mitigate, delay the manifestation, and eventually revert and cure this serious health problem that is already affecting tens of thousands of people around the world. In this section, we will discuss several studies that are key to understanding this devastating disease; we will also use the abbreviation “*htt*”, instead of “*HTT*” (human huntingtin gene), to properly refer to and distinguish the *Drosophila* huntingtin gene and htt to differentiate its protein counterpart.

### 7.2. Transgenic Flies for the Study of HD Initiation and Development

A critical phase in HD pathogenesis is the cleavage of the full-length htt protein, which releases N-terminal fragments of variable sizes and has a polyQ stretch that becomes cytotoxic in neuronal cells [94]. A recent study using a transgenic fly model (*FL-HTTQ200*) (for HD fly models’ information, see Table 1) expressing the tobacco etch virus (TEV) showed the proteolytic consequences of full-length htt. N-terminal hydrolysis disrupts intramolecular interactions within htt, causing toxicity in flies. In addition to canonical pathogenic N-terminal fragments, C-terminal fragments can also be created by double proteolysis, and both the N-terminal and C-terminal byproducts induce greater toxicity via dilation of the ER and increased ER stress, thus impairing dynamin 1 protein activity and leading to cell death. Upon TEV induction, the double proteolysis of htt was significantly more toxic to flies than any other combination. Thus, N-terminal and C-terminal metabolites of htt proteolysis are toxic *in vivo* [95].

When studying HD development, it was found that one of the earliest signs of neurological deterioration in the progression of this disorder is the sleep cycle. Sleep disruptions are common in pre-symptomatic patients and early HD bearers, and include altered sleep architecture, interrupted sleep–wake behavior or sleeplessness with reduced rapid eye movement sleep [REM], increased sleep latency, and increased nocturnal activity [96]. Sleep defects may reveal the initial pathological development of HD; flies expressing the huntingtin mutant version exhibit decreased nighttime sleep in early adulthood. In this regard, the *Drosophila* model system has been critical for the molecular and genetic dissection of the circadian clock, and has emerged as a powerful system for understanding sleep regulation [96]. A study was conducted using the transgenic HD fly model expressing the full-length mutant htt protein (*p upstream activating sequence [UAS]-Htt128Q*), and three different genomic inserts of an N-terminally truncated mutant *htt* transgene to identify disturbances during sleep-wake behavior. In general, the elevation of cyclic adenosine monophosphate (cAMP), protein kinase A (PKA), and cAMP response element binding (CREB) activity decreases sleep in flies. Genetic diminution of PKA signaling suppresses HD-related larval lethality and reverses sleep and activity phenotypes, complementing the neuronal mutant *htt* expression. In general, the PKA reduction prolongs the median lifespan of the HD model flies [96]. Two different methods were used to genetically alter PKA levels, namely: (1) by means of upstream activating sequence promoter (UAS-driven) transgenes that express ribonucleic acid interference (*RNAi*) directed against the catalytic subunits of PKA, knocking down its activity; or (2) by the overexpression of a regulatory subunit. Both methods suppress lethality because of the directionality of the altered PKA activity, and not the off-target effects of *RNAi* [97]. Decreasing PKA signaling partially or fully suppresses all of these phenotypes, suggesting a relationship between aberrant signaling, sleep, and early disease progression. The early onset of these sleep defects suggests that they may be a systemic readout of primary molecular dysfunction in HD, and cAMP/PKA could be established as prevenient indicators of disease and serve as potential therapeutic targets for intervention [96,97].

### 7.3. Protein Targets as a Potential Treatment of HD Using the Drosophila Model

#### 7.3.1. Transcriptional Factors

Other proteins and genes have been explored as possible drug targets, including the transactivation domain (TAD) of a self-regulated transcription factor codified by the *c-Myc* gene. The tissue-specific upregulation of *Drosophila c-Myc* (*dmyc*) dominantly suppresses polyQ-mediated toxicity in an HD *Drosophila* model (*UAS-mRFP.Htt.138Q*), mostly by improving histone acetylation and re-establishing the global transcriptional impairment [98,99]. In humans, the *c-myc* gene is translated into three distinct isoforms that produce three corresponding protein products, namely: c-Myc1, c-Myc2, and c-MycS. C-MycS has an unusual form of regulation, as it acts as a dominant negative suppressor of c-Myc1 and c-Myc2 functions. Recently, it was proposed that, similar to *dmyc*, the targeted expression of human *c-myc* in flies also suppresses polyQ-induced neurodegeneration via an analogous mechanism [99]. Furthermore, the TAD of human c-Myc appeared to be critical for the rescue event. It was determined, using multiple *Drosophila* transgenic lines that independently expressed three different transcript isoforms encoded by human *c-myc*, that similar to its *Drosophila* homologue (*dmyc*), the targeted expression of human *c-myc* suppresses polyQ-mediated neurotoxicity, both phenotypically and functionally. Therefore, c-Myc is a promising target for the design of novel drug molecules against HD [99].

Transcriptional dysregulation is one of the mechanisms underlying polyQ diseases and can be partially explained by the fact that HTT binds to several transcription factors. Mutant *htt* binds to p300, CREB binding protein, and Pcaf, inhibiting their activities mainly as a result of decreased histone acetylation. The range of histone acetyltransferases (HATs) interacting with mutant *htt* and, consequently, the reduction in HAT activity, have been evaluated using *Httex1pQ93*, a *Drosophila* transgenic model of HD generated by standard P element-mediated transformation [100]. A decrease in Pcaf (a complex involved in DNA repair) leads to a reduced average of rhabdomeres per ommatidium in the flies’ expression a mutant *htt* and a Pcaf null allele, compared to the control sibling flies only expressing a mutant *htt*; indicating that reduced Pcaf levels enhance neurodegeneration [101]. Interestingly, the eclosion of the flies expressing Pcaf and *htt* simultaneously resulted in a non-significant higher degeneration than the sibling controls expressing the mutant *htt*. Additionally, the soluble Pcaf protein is altered in *Httex1pQ93* flies and the mutant *htt* expression is not reduced; taken together, these results indicate that the reduction of Pcaf has a significant impact on HD pathology, however, therapeutic strategies to elevate the Pcaf levels are ineffective in ameliorating HD pathology [100,101]. The overexpression of Pcaf, however, probably requires other additional factors, in order to effectively increase the level of soluble Pcaf in *Httex1pQ93* [101].

#### 7.3.2. Autophagy and Cargo Recognition

The main function of the vertebrate HTT protein in the cell has not been fully elucidated. As previously mentioned, reports suggest that HTT has a dual function as a scaffold in selective autophagy, by promoting cargo recognition and autophagy initiation [103]. Physiologically, autophagy is regulated in the healthy cell; it is a destructive mechanism that disassembles unnecessary or dysfunctional constituents during cell processes [104]. Defective autophagy in HD-affected neurons is exhibited by the presence of empty autophagosomes and promotes cargo protein failure. To understand this process, one study used homozygous *Drosophila* mutants lacking the *htt* gene (*dhtt-ko*) and tested the physiological function of *htt*, tracking the ectopic expression of a truncated form of the microtubule-binding protein Tau (Tau-ΔC) [103]. This resulted in a prominent collapse of the thorax in *dhtt-ko* flies as a result of severe muscle loss, and an accelerated decline in mobility and lifespan, which was not observed by the Tau expression alone. These phenotypes were fully rescued by the *dhtt* genomic rescue transgene. Furthermore, the expression of human HTT in *dhtt-ko* flies rescued both the mobility and longevity defects of *dhtt-ko* mutants and partially rescued the Tau-induced morphological and behavioral defects of *dhtt-ko* flies, suppressing most of the autophagic defects observed in *dhtt-ko* [103].

#### 7.3.3. The Aggregation Mechanism

There is evidence that the htt protein aggregates can spread between neurons [105]. Although there is information on how proteins pass from one cell to another in this disseminating protein mechanism, the specific processes remain unclear. There is some evidence indicating that this occurs through synaptic connections, tunneling nanotubes, or by means of exosome machinery using the *UAS-mRFP.Htt.138Q* HD fly model with the *Or83b-Gal4* driver to target the expression into the axonal terminals of the antennal lobe, where the htt protein aggregates are located at the synaptic terminals in the antennal lobe of the *Drosophila* central brain when expressed in olfactory receptor neurons. These aggregates start to spread to other brain regions. In order to test if this phenomenon is unique to the toxic polyQ form, a construct with a much shorter polyQ tract was used as a control, *UAS-mRFP.Htt.15Q*, which does not form aggregates and therefore does not spread through the brain [106]. The underlying mechanism by which htt extends throughout the brain involves SNARE (SNAP [Soluble NSF {N-ethylmaleimide-sensitive factor} Attachment Protein] REceptor) machinery; this was shown by knock down *N*-ethylmaleimide-sensitive fusion protein 1 (NSF1) using the *UAS-comt^RNAi^* method, as NSF1 is required for SNARE disassembly and for complex recycling, as well as for lysosomal trafficking and autophagy. This strategy showed a significant decrease in htt spreading. Similar results were observed when interfering with the dynamin function, confirming that htt aggregate release requires dynamin and NSF1 activity through a SNARE mechanism and its insertion into other neurons by active endocytosis [107].

#### 7.3.4. The Fly’s Eye as a Tool for the Study of HD

Considering the previous data, suppressing the toxicity generated by the polyQ mutation in htt should be a fundamental target. A pioneer study using the *Drosophila* HD model and other polyQ diseases (*GMR-GAL4UAS-127Q*) was performed using transposable elements affecting the fly’s eye, in order to screen for genetic factors modifying the degeneration caused by the polyQ expression [102]. Among the 7000 P-element insertions, several suppressor strains were isolated, two of which led to the discovery of the suppressor genes. The first strain is the predicted *dHDJ1* product, which is homologous to the human HEAT shock protein 40lHDJl. The second strain, dTPR2, is homologous to the human tetratricopeptide repeat protein 2. Each of these proteins contains a chaperone-related J domain. The suppression of the polyQ toxicity was verified in transgenic flies, which had severe externally evident eye aberrations, and were used to screen for dominant modifiers of the polyQ repeat toxicity by examining the genes in the vicinity of a series of P-element chromosomal insertion sites. The abnormal eyes of the transgenic flies dramatically improved in the presence of the suppressor P-element insertion. With this insertion, the eye preserved its globular structure, pigmentation, and a uniform bristle arrangement [102]. Reports like this create higher hopes for the development an effective treatment to stop HD progression. Another encouraging study describes the potential of curcumin ([1E,6E]-1,7-bis [4-hydroxy-3-methoxyphenyl]-1,6-heptadiene-3,5-dione), a polyphenolic compound with an outstanding safety profile. Curcumin is the major bioactive component of turmeric, a phytochemical and an ingredient commonly used in Asian cuisine and medicine. Recent studies have discovered that curcumin is a positive therapeutic compound, given its strong antioxidant, anti-inflammatory, and anti-protein aggregation effects [108]. Curcumin suppresses degeneration of photoreceptor neurons (indicative of disrupted internal eye architecture) in transgenic *Drosophila* expressing the exon1 fragment of mutant *htt* with *48* or *93* glutamine residues (*httex1pQ93* and *Q48* peptides) [109,110]. Curcumin also ameliorates the extensive degeneration and external dysmorphology of the eye, caused by the expression of *Httex1pQ93* in all of the cells of the *Drosophila’s* eye [110]. This progressive loss of photoreceptor cells expressing *httex1pQ93* and *Q48* peptides can be observed quantitatively (as a measure of neurodegeneration) with the pseudopupil technique [111]. Nurturing the fly *httex1pQ93* and *Q48* with curcumin-supplemented food was enough to suppress the photoreceptor neuron degeneration in a dose-dependent manner. The most significant suppression was achieved at a fed dose of 10 μM, showing that dietary curcumin suppresses polyQ-induced neurodegeneration and internal morphological defects of the eye, with no side effects. Reports also found a substantial reduction in polyQ-induced cell death in third-instar eye discs, due to the administration of curcumin, which has been implicated in alleviating cytotoxicity, oxidative stress, and apoptotic cell death in neurological disorders [110]. A fact to thoroughly consider is that the prevalence of neurodegenerative diseases among people living in the Asian subcontinent, where spices are regularly consumed, is lower than in Western countries (see [38]). Dietary habits could be a critical factor for producing significant improvements in HD prevention and treatment [110].

#### 7.3.5. Metabolic Imbalance

HD induces imbalances in several metabolites in humans and in non-fly HD models; mutant huntingtin interacts directly with metabolites such as valine, alanine, glutamine, and glycerol [112]. By means of nuclear magnetic resonance spectroscopy in a *Drosophila* HD model (*UASHTT-EX1-PQ93/CyO*), alterations in metabolomics were detected in several phenotypes expressing htt with different numbers of polyQ repeats, showing mainly perturbations in the development of classified adult ommatidia; these transgenic lines carried 20 (control), 93 (intermediate), and 127 (diseased) polyQ repeats. The main result of the metabolomic profile in the HD flies was that the major metabolite imbalance included nicotinamide adenine dinucleotide (NAD), lactate, and pyruvate, compared to the control profile (20 polyQ). Interestingly, these metabolites are related to the metabolic pathways involved in cellular energetics. In particular, decreased NAD levels could be associated with cell death (a hallmark of HD) as a result of a deficit in cellular energy supply. Additionally, an increase in the lactate/pyruvate ratio compared to the control suggested mitochondrial dysfunction. This observation is similar to the increased pyruvate/lactate ratio found in HD patients. Based on the above information, the imbalance in the NAD and lactate/pyruvate ratios are obvious perturbations in cellular energetics of HD fly tissues [113].

#### 7.3.6. Transport Proteins and Trafficking

Rab GTPases constitute the largest family of small GTPases; they are highly active in neurons and are key regulators at intracellular membrane trafficking. Different Rab GTPases are involved in early transport vesicle formation through membrane-vesicle interaction and are located in the ER-Golgi, early endosomes, late endosomes, and synaptic vesicles [6,114]. *Drosophila* native htt seems to participate in axonal transport, in the movement of several Rab proteins, and in the trafficking of Rab-containing vesicles, thus impairing the anterograde and retrograde motility when htt is expressed in reduced amounts, and stimulating the anterograde motility of Rab2—a GTPase linked to its corresponding mammalian Rab [80]. At least 60 different Rab GTPase family members are present in the human genome; meanwhile, in *Drosophila* there are only 26 members. The Rab5 GTPases are regulators of intracellular membrane trafficking in early endocytosis and they also play a role in autophagy [115]. In HD, Rab5 GTPases mediate the degradation of the mutant htt protein, which contains an abnormally long polyQ tract. Interestingly, in mammalian cells (COS-7 and MEF cells), Rab5 activity can decrease the polyQ toxicity and its aggregation. Furthermore, Rab5 overexpression can rescue the typical rhabdomere degeneration in photoreceptors in the *Drosophila* HD model (*CyO/If; UAS-Rab5-EGFP-elavGal4/MKRS*). Additionally, protein interactions showed that Rab5 is a member of a complex that includes the proteins, Beclin 1 and PI 3-Kinase Vps34, which regulate the early steps of autophagosome formation. The 3MA treatment (a PI-Kinase inhibitor) and siRNA knockdown of Vps34 had similar effects on the Rab 5 loss of function; the autophagosomal precursors accumulated, whereas autophagic vacuoles decreased. This accumulation of precursor structures was observed in response to Rab5 inhibition. Otherwise, Vps34 might block the progression from early autophagosomal structures to mature autophagic vacuoles [116].

Morphological studies in one of the first HD mouse models, the R6/2, with 116 to 150 CAG repeats, manifested a behavioral phenotype very similar to human HD [56], indicating a clear decrease in the density of MSSN the excitatory synaptic contacts, in addition to a significant reduction of the postsynaptic spine terminal [117]. In a *Drosophila* HD model, the accumulation of pathogenic huntingtin protein (*UAS-mRFP.Htt.138Q*) in motor-neuron terminals changed the synaptic morphology, thus altering axonal branching and leading to neuromuscular junction overgrowth in a dose-dependent manner. Furthermore, this excessive growth of synaptic connections induces pupal lethality in most cases. The synaptic overgrowth phenotype is related to abnormal endocytosis and endosomal traffic in the *HttQ138* model. This mutant huntingtin exhibits protein–protein interaction with Sorting Nexin 16 (Snx16), a transport protein with a critical role in intracellular protein trafficking from early to late endosomes. It also regulates the trafficking of bone morphogenetic protein (BMP) receptors, which belong to the transforming growth factor-beta (TGF-beta) superfamily. *HttQ138-Snx16* interaction in early endosomes disrupts normal endosomal trafficking and increases the size and number of endosomes that upregulate the BMP signaling in the nerve terminals of flies, thus triggering a robust overgrowth not only at the axon level, but also of the smaller synaptic bottoms or axonal branches [118].

#### 7.3.7. Heat Shock Proteins

One of the most common pathological features of HD is protein misfolding and the accumulation of mutant proteins as insoluble aggregates. The HEAT shock response (HSR) is a highly conserved mechanism in order to maintain cellular integrity. HSR transcribes multiple chaperones, including heat shock proteins (HSPs), where HSF1 is the principal transcription regulator of HSR components, and HSP90, which is known for repressing HSF by itself or associated with a multichaperone complex [119]. To determine whether the HSR mechanism had been lost in both the murine and fly models of HD, the transcription, expression, and phosphorylation of HSF1 were analyzed. The HSF1 levels decreased in HD and clearly increased upon chronic dexamethasone treatment, a synthetic glucocorticoid, in different brain regions of the HD models and, interestingly, in the control animals. These findings suggest that the dexamethasone induced HSF1 transactivation in HD as well as in wild-type animals. Dexamethasone reduces motor symptoms and protein aggregates in the neostriatum, cerebellum, and cortex in an HD murine model [120] and in *Drosophila* eye imaginal discs of larvae in an HD fly model (*UAS-HSF1-RNAi*) [121]. The effect of dexamethasone in the HSF1 induction was confirmed with an HSF1 RNAi. The mechanism by which the dexamethasone treatment induces HSF1 upregulation was associated with the down-expression of HSP90 in HD 150Q cells, HD mice, and in a fly model; suggesting anti-inflammatory drugs, such as dexamethasone, as a potential pharmacological treatment of HD and other polyQ disorders [121].

#### 7.3.8. mTOR Pathway Inhibition

In cell lines, inhibitors of the autophagy–lysosome pathway impair the alterations in the mutant htt fragment turnover. Previously, autophagy induction was effective at protecting against the toxic effects of mutant htt by means of pharmacological treatment with rapamycin, a specific inhibitor of *mTOR*, which in turn inhibits autophagy activity [122]. Rapamycin reduces protein aggregate formation and cell death only in the early hours of post-transfection, suggesting that inducing early rapamycin autophagy in vivo may attenuate HD effects against the toxicity of htt accumulation. Remarkably, mTOR is sequestered into htt aggregates; this interaction was confirmed by chimeric proteins, tissue immunolabeling, and Western blotting, as well as by a co-immunoprecipitation assay, suggesting that this strong interaction could alter the nucleocytoplasmic transport that is crucial for mTOR activity. To corroborate this, the mTOR activity was inferred by phosphorylating 4E-BP1 and S6K1, two downstream proteins of the mTOR pathway. Phosphorylation was reduced in mutant cells with aggregates, and no variations were observed when adding rapamycin. However, rapamycin inhibited the S6 activity in the control cells, and decreased the rhabdomere neurodegeneration was observed in the fly model (*gmr-Htt[exon1]Q120*). This implies that mutant *htt* causes translational deregulation by altering the mTOR signaling pathway. The results suggest that only early treatment with rapamycin can attenuate the HD characteristics, by inducing autophagy mediated by mTOR inhibition [123].

#### 7.3.9. Oxidative Stress Associated to HD

As previously mentioned, htt is a ubiquitous protein spread throughout the body during all stages of life. Interestingly, HD patients exhibit an important prevalence of cardiac failure as a concomitant pathology. Using a *Drosophila* model (*Httex1pQ93*) of cardiac amyloidosis that exhibited accumulation of htt protein with different polyQ repeat lengths (PolyQ-25; PolyQ-46; PolyQ-72; PolyQ-102), it was determined that longer polyQ repeats (PolyQ-72 and PolyQ-102) in fly hearts were a molecular factor for cardiac dilation and reduced heart contractility [107]. Moreover, fly hearts with longer polyQ exhibited reduced myofibrillar content and severe myofibrillar disorganization; and subsequently, the accumulation of toxic aggregates and severe mitochondrial fragmentation that induced an increase in oxidative stress in PolyQ-72 and PolyQ-102, as demonstrated with the superoxide indicator dihydroethidium. To verify the role of oxidative stress, the superoxide dismutase (SOD1) enzyme was overexpressed and treated with the antioxidant agent, Resveratrol, which partially suppressed the cardiac dysfunction induced by the polyQ repeats. Likewise, the overexpression of UNC-45, a chaperone necessary for myosin folding and accumulation, also improved heart contraction and heart rate regularity, suggesting that polyQ protein misfolding plays a role in mitochondrial dysfunctions and subsequent oxidative stress, inducing cardiac disease [124]. Finally, metal-induced oxidative stress, in particular copper-dependent toxicity, was shown to be an important component of disease progression in a *Drosophila* model of HD [125].

Oxidative stress is an outstanding feature to consider in HD. When screening a yeast open reading frame collection, antioxidants that suppressed *htt103Q* toxicity were identified. The antioxidant genes of glutathione peroxidase *GPx1* and *GPx2* are overexpressed in a cell model of mutant *htt*, suppressing *htt103Q* toxicity and decreasing caspase (a protease enzyme family with an essential role in inflammation and programmed cell death) activity; whereas *mGPx1* provides neuroprotection in the eyes of HD flies and restores decreased locomotor activity and circadian arrhythmia in a *Drosophila* HD model. Similar observations in decreasing the htt toxicity were reported when using Ebselen, a mimic molecule of glutathione peroxidase, widely used in clinical practice against strokes. It is known that oxidation contributes to the chronic development of this pathology; however, the overexpression of other antioxidants, such as superoxide dismutase, catalase, and glutathione reductase, do not protect against *htt103Q*-mediated toxicity [126].

#### 7.3.10. The Kynurenine Pathway

The kynurenine pathway metabolite kynurenic acid (KYNA) is a modulator of cholinergic and glutamatergic neurotransmission. KYNA production is dependent on kynurenine 3-monooxygenase (KMO) [127]. Several neurodegenerative disorders are related to alterations in the kynurenine pathway (KP) of tryptophan degradation. KP contains metabolites shown to be neuroactive, namely: KYNA (neuroprotective) and 3-hydroxykynurenine (3-HK) (neurotoxic). In *htt93Q*, a fly model of HD, the 3-HK/KYNA ratio increases, supporting a pathogenic role for KP. To explore the effects of KP manipulation in this HD fly model, the branching point between the 3-HK and KYNA synthesis was modulated by KMO. The genetic interruption of KMO with a mutant (cn3) showed a 26% rescue in the photoreceptor neurons on the first day (rhabdomeres) and a 47% rescue on the seventh day; whereas with RNAi, the genetic interruption of KMO showed a 65% and 67% rescue. Additionally, in both cases, the 3-HK and 3-HK/KYNA ratio decreased. Regarding a potential pharmacological modulator, UPF 648, a KMO inhibitor, was tested in *Htt93Q*; 90% of the flies fed with this inhibitor rescued and increased KYNA, and the 3-HK/KYNA ratio subsequently decreased, supporting the idea that the KMO inhibitor drugs reduce the neurodegenerative effects of HD. Finally, when the *Htt93Q* cn3 flies were fed with 3-HK, a neurodegenerative process was restored, and when the flies were fed with KYNA, a protective effect was observed, supporting the neuromodulator effect of 3-HK and KYNA. These findings led to the conclusion that the metabolites of the KP pathway modulate neurodegeneration and thus have the potential to be used as a pharmacological therapy [127]. It should be noted that the commonly used white *Drosophila* mutant is defective in 3HK accumulation. An issue to be considered when studying the current *Drosophila* models of HD is that the white mutant has been used as a convenient marker for transgenesis [43].

### 7.4. The Glia; Studying an Almost Unknown Factor Involved in HD Pathogenesis Using the Drosophila Model

Despite efforts to define the role of glial cells in HD pathogenesis [128,129,130], or the effects that abnormal polyQ repeats have on glia [131], these aspects remain almost unattended in HD. Glial cells express huntingtin constitutively; the aggregation of mutant htt in glia induces locomotor deficits and decreases lifespan in the fly HD model [132,133]. This model (*DmGluT1*), with locomotor deficits and early lethality, could be rescued by the overexpression of *DmUCP5*, a homologue of mitochondrial uncoupled protein 5 (*UCP5*) in humans; generating, for this reason, a *Drosophila DmUCP5* transgenic line [134]. Protein aggregation in glial cells can cause dysfunction in both neurons and glia, as it is intrinsically cytotoxic and negatively influences the nearby neurons. The activation of glial phagocytosis is one way by which the aggregates or damaged neurons are cleared from the CNS [135]. The *UAS-GAL4* system is a powerful tool that has been used in *Drosophila* as a selective strategy to express mutant htt in glial cells so as to identify the htt implications [132]. In a recent report, the *Drosophila* model system was established to assess the role of phagocytic glia in the uptake and clearance of polyQ aggregates formed by a pathogenic fragment of htt in neurons within an intact CNS. The report determined that the mutant htt aggregates localized in neurons could be eliminated by glia through a phagocytic process that requires the cell surface engulfment receptor, Draper [136]. Phagocytosed neuronal htt aggregates can access and initiate a prion-like assimilation of normally soluble, wild-type htt proteins in the glial cell cytoplasm. These findings suggest that the phagocytic clearance of neuronal htt protein aggregates by glia might contribute to the spreading of pathogenic protein aggregates in neurodegenerative disease. Moreover, these findings have important implications for the potential role of glia as a fundamental mediator of protein aggregation in the suppression and/or progression of neurodegenerative diseases [136]. Abnormal PolyQ expansion in the blood-brain barrier and blood-retina barrier glia impaired both barriers and reversed the polarity in electroretinography. Moreover, polyQ repeats also restrict fly lifespan and reduce the transcriptional levels of Repo, a transcriptional factor necessary for glia differentiation. Otherwise, the expression of chaperones HSP40 and HSP70, a bi-complex protein that regulates adenosine triphosphate, partially rescued the shortened lifespan caused by htt-expanded proteins, suggesting that polyQ aggregates may affect the normal expression of transcriptional factors like Repo, which is rescued by HSP chaperones, therefore inhibiting htt protein aggregate formation [131].

## 8. Concluding Remarks

**Figure 3:**
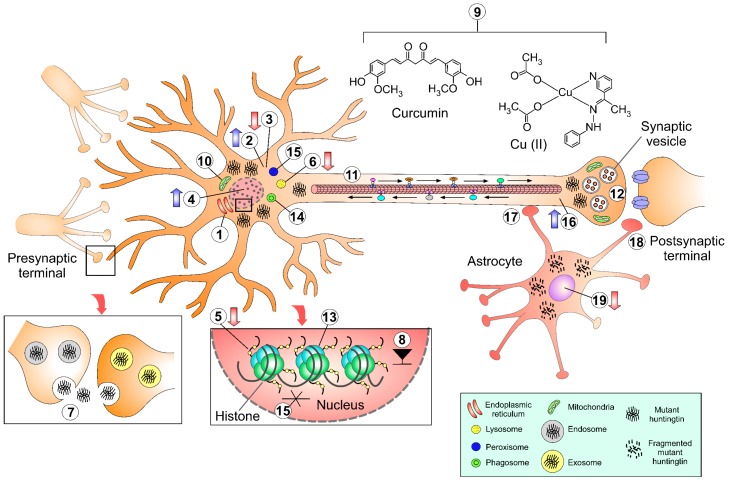
Some examples of the legacy of the fruit fly in solving molecular questions in Huntington’s disease. Numbers are related to the conclusions of this review. Upwards arrows (blue) indicate increasing expression. Downwards arrows (red) indicate decrease in expression.

In conclusion, a summary of the HD changes and the consequences of protein aggregation mentioned throughout the text using HD fly models are depicted in Figure 3.

**1**. A double proteolysis of both the N-terminal and C-terminal becomes highly detrimental for the dynamin 1 protein, leading to reticulum stress and malfunction. 

**2**–**3**. The increase of cAMP and cAMP/CREB produce early onset sleep defects, inducing an increase in PKA signaling. Decreased cAMP/CREB activity could partially attenuate sleep alterations. Both cAMP and PKA levels could be established as indicators of disease and/or therapeutic targets.

**4**. *Homo sapiens* and *Drosophila melanogaster c-Myc* suppress polyQ toxicity by improving histone acetylation. Re-establishing the transcriptional impairment of c-*Myc* could be a novel pharmacological target for HD. 

**5**. Mutant htt binds to Pcaf (a complex involved in DNA repair), inducing a decrease in the histone acetylation and Pcaf levels, and worsening the neurodegenerative process. 

**6**. Defective autophagic activity was exhibited by the presence of empty autophagosomes, promoting failure of the cargo proteins.

**7**. Dissemination of htt protein occurs because of synaptic connections, tunneling nanotubes, or by means of exosome machinery. Release of mutant *htt* requires dynamin and NSF1 activity through NSF attachment to protein receptor SNARE, and its recapture into other neurons occurs by active endocytosis. 

**8**. Using P-element insertions, two suppressor genes were discovered (*dHDJ1* and *dTPR2*, with their respective *Homo sapiens* homologous). Both insertions dramatically decreased the aberrations caused by polyQ toxicity. 

**9**. Curcumin-supplemented food and copper chelation ameliorate polyQ degeneration and cell death. 

**10**. Major metabolite imbalances include lowered NAD (linked with cell death due to a deficit in cell energy) and an increased lactate/pyruvate ratio, suggesting mitochondrial dysfunction. 

**11**. Native htt in *Drosophila* seems to participate in axonal transport, Rab protein movement, and trafficking of Rab-containing vesicles. Additionally, in the presence of mutant *htt* Rab5 (a regulator of intracellular membrane trafficking), a protein with a key role in autophagy activity, which decreases as a consequence of polyQ toxicity and aggregation. Overexpression of Rab5 rescues cells from degeneration. 

**12**. Pathogenic htt accumulation in neuron terminals changes synaptic morphology by altering axonal branching, thus leading to neuromuscular junction overgrowth. The htt-Snx16 interaction disrupts normal endosome trafficking and has a general increase in the endosomal size and number. 

**13**. Expression levels of HSP90 and HSF1, a transcription regulator of HSR components, decrease in HD. However, these levels increase upon chronic dexamethasone treatment, which improves motor symptoms and reduces protein aggregates. 

**14**. Autophagy induction was effective at protecting against toxic effects of mutant *htt* by applying pharmacological treatment with rapamycin, a specific inhibitor of *mTOR*, which in turn inhibits autophagic activity. 

**15**. Overexpression of the antioxidant glutathione peroxidase *GPx1* gene during HD helps to suppress htt toxicity, as well as to decrease specific caspase activity providing neuroprotection and restoring decreased locomotor activity and circadian arrhythmia. 

**16**. The kynurenic pathway (KP), is a modulator of cholinergic and glutamatergic neurotransmission. Several neurodegenerative disorders have been associated with alterations in KP. Dysregulation of KP produces toxic metabolites that can damage several neurons. Flies fed with UPF 648, a KM regulator, rescue 90% of the neurons from damage. UPF 648 has the potential to be used as a pharmacological therapy. 

**17**. The phagocytosed neuronal htt aggregates can access and initiate prion-like assimilation in the glial cell cytoplasm, which might contribute to spreading pathogenic protein aggregates. 

**18**. Mutant htt aggregates expressed in neurons could be eliminated by glia through a phagocytic process that requires the cell surface engulfment receptor, Draper.

**19**. Abnormal polyQ repeats also restrict fly lifespan and reduce transcriptional levels of Repo, a transcriptional factor necessary for glia differentiation.

## 9. Insights and Directions for Future Research

The *Drosophila* model has been used over many years to study several of the consequences induced by polyQ disorders linked to HD, because of the simple fact that *Drosophila* huntingtin does not express an expanded polyQ sequence in its amino terminal domain. However, the following imperative question remains: What have we learned about the disease, taking advantage of the uniqueness of the fruit fly compared with other animal models, and how can we use this knowledge to enrich future research in order to treat this disease more effectively. In order to answer to this question, we propose to increase the research focused on the integrative process using transgenic flies expressing defective interacting proteins, and the mutant polyQ human htt, followed by the validation of behavioral experiments. These model flies can be treated with new molecules, with the aim of developing more effective and selective drugs than the ones that are currently being used. These molecules can be addressed to one or more of the multiple targets potentially involved in the development of the disease. Among these targets are the following: partially stopping the double proteolysis of both htt in the N-terminal and C-terminal; downregulating the cAMP and PKA levels in specific pathways; upregulating *c-Myc* levels by alternative methods; downregulating or stopping the binding of htt to Pcaf, avoiding decreased histone acetylation; targeting and destroying empty autophagosomes and their damaged cargo proteins; downregulating or stopping the interaction of htt with dynamin, NSF1, or the protein receptor SNARE by endocytosis; downregulating or stopping the *htt-Snx16* interaction, which disrupts normal endosome trafficking; downregulating or suppressing the expression of the antioxidant glutathione peroxidase *GPx1* gene; and upregulating the levels of Repo, a transcriptional factor necessary for glia differentiation, which is reduced during HD. Also, there are very promising perspectives for performing more extensive research in subjects such as the discovery of genes *dHDJ1* and *dTPR2*, with their respective human homologous; the curcumin molecule in the treatment of polyQ degeneration; the major metabolite imbalance as a consequence of HD; the overexpression of Rab5 (a regulator of intracellular membrane trafficking), and its role in rescuing cell degeneration; some treatments based on dexamethasone, rapamycin, and some inhibitors of the kynurenic pathway, which have been very promising for improving motor symptoms, reducing protein aggregates, and inhibiting autophagic activity. Lastly, a better understanding of some related cellular mechanisms could produce key solutions for HD, for example, understanding prion-like assimilation and the phagocytic process of glia. All of these perspectives offer great possibilities for future lines of research in order to better understand and treat HD, and which would not be possible without the use of the *Drosophila* model and its extraordinary potential when using tools for gene expression, such as the *GAL4/UAS* system, *UAS-RNAi*, P-elements, genetics mosaics, and CRISPR.

## Figures and Tables

**Table 1:** *Drosophila* mutants named in Section 7. UAS—upstream activating sequence; UCP—uncoupled protein; *HTT*—huntingtin; CAG—cytosine–adenine–guanine; HD—Huntington’s disease; * Referenced in text as.

· Allele Symbol (* Referenced in text as)	Alternative Names	FlyBase ID	General Description
· Hsap\^HTT200Q.UAS^ * *FL-HTTQ200*	*Hsap\^HTT200Q.Scer\UAS^* *Hsap^HTT200Q.UAS^*	FBal0323497	UAS regulatory sequences drive the expression of a full-length Hsap\*HTT*, containing an expanded 200 polyQ repeat. Induces rapid age-progressive decline of locomotor abilities in adult flies. Progressive defects of locomotor behavior.
· Hsap\^HTTQ128.Scer\UAS^ * *pUAS-Htt128Q*	*UAS-Htt-Q128, Htt-Q128, UAS-HttQ128, UAS-Htt^548aa^-128Q, Hsap\^HDQ128.Scer\UAS^, Hsap\HTT^Q128.Scer\UAs^, HttQ128*	FBal0156385	Scer\UAS sequences drive expression of the N-terminal 548 aa of the Q128 Hsap\HD cDNA, which encodes the pathogenic protein. Defective for behavior, circadian rhythm, eye color, locomotor behavior, and neuroanatomy. Reduced photoreceptor depolarization and complete abolishment of synaptic transmission in response to light. Cytoplasmic Hsap\HD aggregates are seen in neurons and non-neural tissues, aggregates are transported in larval motor axons, and accumulate in presynaptic neuromuscular junction terminals. Uncoordinated movement and abnormal grooming behavior. Premature death.
· Hsap\HTT^Q93.ex1.Scer\UAS^ * Httex1pQ93 * UASHTT-EX1-PQ93/CyO * UAS-Htt exon1-Q93 * *Htt93Q*	*UAS-Httex1p Q93, Httex1p Q93, UAS-htt exon-1-Q93, UAS-Httex1p-Q93, P{UAS-Httex1p Q93}, Htt93Q, Httex1-93Q, UASHTT-EX1-PQ93, HttEx1Q93, P{UAS-Httex1p Q93}4F1*	FBal0127292	Leads to an obvious loss of one or more photoreceptors, leading to a disorganization of ommatidia, exhibiting a progressive loss of vision. Results in the formation of aggregates in larval eye imaginal discs and subsequent age-dependent retinal degeneration and visual impairment. Neural degeneration and alteration of the diameter of synaptic vesicles, accumulation of organelles is seen in neurons producing a defect in axonal transport, increasing cell death. Initially hyperactive with gradual loss. Reduces mobility and lifespan.
· HsapHTT^Q138.Scer\UAS.T:Disc\RFP-mRFP^ * *UAS-mRFP.Htt.138Q*	*Hsap\HTT^Q138.Scer\UAS.T:Disc\RFP-mRFP^*	FBal0267405	Containing a pathogenic tract of 138 polyQ repeats. Defects on grooming behavior, locomotor behavior, neuroanatomy, and increased cell death in the central brain and optic lobes. Neurons display morphological indicators of reduced neuronal health, including smaller neuromeres, increased branching, and reduced axonal connectivity. Premature death.
· UAS-mRFP.Htt.15Q * *UAS-mRFP.Htt.15Q*	*Hsap\HTT^Q15.Scer\UAS.T:Disc\RFP-mRFP^*	Control for UAS-mRFP.*Htt*.^138Q^	Non-expanded human *HTT* control.
· GMR-GAL4UAS-127Q * *GMR-GAL4UAS-127Q*	*Zzzz\CAG^127Q.Scer\UAS.T:Ivir\HA1^*	Information in the literature [102]	Containing the expanded 127-CAG repeat. Severe abnormal eyes.
· Hsap\HTT^GMR.Q120^ * *gmr-Htt(exon1)Q120*	*gmr-HttQ120* *gmr-Htt-Q120* *gmr-Q120* *Q120* *gmrHtt(exon1)Q120* *GMR-HD.Q120* *GMR-HTT.Q120* Between others	FBtp0010067	Expression of Hsap\HD amino acids 1–170, with 120 CAG glutamine repeats is governed by the glass multiple reporter (GMR) promoter. Results in neurodegeneration and the loss of rhabdomeres. Show a progressive decrease in the number of visible rhabdomeres per ommatidium. The eyes of flies expressing this allele progressively degenerate.
· Dmel\Rab5^Scer\UAS.T:Avic\GFP-EGFP^ * *CyO/If; UAS-Rab5-EGFP-elavGal4/MKRS*	*UAS-GFP-Rab5* *Rab5-GFP* *UAS-Rab5-GFP* *GFP-Rab5* *UAS-GFPRab5* *UASrab5-GFP* *UAS-GFP:Rab5*	FBal0182041	Modeled by *Hsap\HTTGMR.Q120*. Flies expressing this allele show trichome polarity defects in the wings, and alterations in the synaptic area under the regulation of Scer\GAL4 does not significantly alter the synaptic area.
· Dmel\Hsf^dsRNA.Scer\UAS^ * *UAS-HSF1-RNAi*	*HSF1 RNAi* *UAS-Hsf.RNAi* *UAS-HSF1-RNAi* *Hsf^dsRNA.Scer\UAS^*	FBal0283110	Defects on neuroanatomy. Shows degeneration including in the eyes.
· Dmel\Glut1^Scer\UAS.cBa^ * *DmGluT1*	*DmGluT1* *Glut1^Scer\UAS.cBa^*	FBal0256734	Defects for locomotor behavior, climbing ability, and reduced lifespan.
· Dmel\Bmcp^Scer\UAS.cBa^ * *DmUCP5*	*DmUCP5* *Bmcp^Scer\UAS.cBa^*	FBal0256733	Defects for locomotor behavior, bang sensitive. Shows glial pathology, neuronal defects. Reduction in life expectancy.
For detailed information of drivers: http://flybase.org/search/disease/#/page/1			

## Supplementary Materials

Supplementary materials can be found at http://www.mdpi.com/1422-0067/19/8/2398/s1.
