# Supplementary material for: The Tiny Drosophila Melanogaster for the Biggest Answers in Huntington’s Disease
Source: Int J Mol Sci. 2018 Aug 14;19(8):2398. doi: 10.3390/ijms19082398 (PMC6121572; doi:10.3390/ijms19082398)
Supplement: Supplementary file 1 [file ijms-19-02398-s001.pdf]

```
CLUSTAL O(1.2.4) multiple sequence alignment


Drosophila      ------------------------------------------------------------    0
Takifugu        MATMEKLMKAFESLKSFQQQQG--------------------------------------    22
Danio           MATMEKLMKAFESLKSFQQQQG--------------------------------------    22
Bos             MATLEKLMKAFESLKSFQQQQQQQQQQQ------QQQQPPP---PPQPPQPPQPPPQAQ-    50
Homo            MATLEKLMKAFESLKSFQQQQQQQQQQQQQQQQQQQQQPPPPPPPPPPPQLPQPPPQAQP    60
Rattus          MATLEKLMKAFESLKSFQQQQQQQQP----------PPQAPPPPPPPPPQPPQPPPQGQ-    49
Mus             MATLEKLMKAFESLKSFQQQQ-QQQP----------PPQAPPPPPPPPPQPPQPPPQGQ-    48


Drosophila      -------------MDKSRSSAYDKFVGFVEQLRNTECSQKQKITCFQQIAECIMSPSLAG    47
Takifugu        -----------------PPTA-EEI--VQRQKKEQATTKKDRVSHCLTICENIVAQSLRT    62
Danio           -----------------PLSA-EEL--VQKQKKDLATTKKDRVTHCLTICENIVAQSLRT    62
Bos             ----PPPQPPPPPPPLGPAAA-EEP--LHRPKKELSATKKDRVHHCLTICENIVAQSLRN    103
Homo            LLPQPQPPPPPPPPPPGPAVA-EEP--LHRPKKELSATKKDRVNHCLTICENIVAQSVRN    117
Rattus          ---------PPP-PPPLPGPA-EEP--LHRPKKELSATKKDRVNHCLTICENIVAQSLRN    96
Mus             ---------PPPPPPPLPGPA-EEP--LHRPKKELSATKKDRVNHCLTICENIVAQSLRN    96
                                    * ::   . .  ::   ::*:::     *.* *:: *:

Drosophila      HINYAAHCGTATNVLLLFCEDVDSVVRMSAEENLNKILRSLEKTRVSRILMDLYGEIKRN    107
Takifugu        SPEFQKLLGIAMEMFLLCSDDSESDVRMVADECLNRIIKALMDSNLPRLQLELYKEIKKN    122
Danio           SPEFQKLLGIAMEMFLLCSDDKESDVRMVADECLNKIIKALMDSNLPRLQLELYKEIKKN    122
Bos             SPEFQKLLGIAMELFLLCSDDAESDVRMVADECLNKVIKALMDSNLPRLQLELYKEIKKN    163
Homo            SPEFQKLLGIAMELFLLCSDDAESDVRMVADECLNKVIKALMDSNLPRLQLELYKEIKKN    177
Rattus          SPEFQKLLGIAMELFLLCSDDAESDVRMVADECLNKVIKALMDSNLPRLQLELYKEIKKN    156
Mus             SPEFQKLLGIAMELFLLCSNDAESDVRMVADECLNKVIKALMDSNLPRLQLELYKEIKKN    156
                  ::    * * :::** .:* :* *** *:* **:::::* .:.: *: ::** ***:*

Drosophila      GNQRSLRICLNLFSYYAPQIKEKHIKWYAVRLLQCMTTISQRKETLLQETLCDFVKHFSR    167
Takifugu        GASRSLRAALWRFAELAHLIRPQKCRPYLVNLLPCLTRITKRQEETIQETLAAAMPKIMA    182
Danio           AASRSLRAALWRFAELAHLVRPQKCRPYLVNLLPCLTRITKRQEETVQETLSSSIPKIMA    182
Bos             GAPRSLRAALWRFAELAHLVRPQKCRPYLVNLLPCLTRTSKRPEESVQETLAAAIPKIMA    223
Homo            GAPRSLRAALWRFAELAHLVRPQKCRPYLVNLLPCLTRTSKRPEESVQETLAAAVPKIMA    237
Rattus          GAPRSLRAALWRFAELAHLVRPQKCRPYLVNLLPCLTRTSKRPEESVQETLAAAVPKIMA    216
Mus             GAPRSLRAALWRFAELAHLVRPQKCRPYLVNLLPCLTRTSKRPEESVQETLAAAVPKIMA    216
                .  **** .*  *:  *  :: :: : * *.** *:*  ::* *  :****.  : ::

Drosophila      HIQQGLSDSESCKLFETFLDQISSDCAVKRRCSAQNCMSLIENARNRSLMARHGVNKVME    227
Takifugu        ALGHFANDGEIKMLLKSFVANLKSSSPTIRRTAASSAVSVCQHSRRTSYFYTWLLNVLLG    242
Danio           ALGHFANDGEIKMLLKAFVANLKSSSPTIRRTAASSAVSVCQHSRRTHYFYTWLLNVLLG    242
Bos             SFGNFANDNEIKVLLKAFIANLKSSSPTVRRTAAGSVVSICQHSRRTQYFYSWLLSVLLG    283
Homo            SFGNFANDNEIKVLLKAFIANLKSSSPTIRRTAAGSAVSICQHSRRTQYFYSWLLNVLLG    297
Rattus          SFGNFANDNEIKVLLKAFIANLKSSSPTVRRTAAGSAVSICQHSRRTQYFYNWLLNVLLG    276
Mus             SFGNFANDNEIKVLLKAFIANLKSSSPTVRRTAAGSAVSICQHSRRTQYFYNWLLNVLLG    276
                 : :  .*.*   *:::*: ::.*.. . ** :* . :*: :::*.   :    :. ::
```

```
Drosophila      LLLTDQQ---ANSVLGALGLLRLLLPQLIRGYPGDSHEDSESLAGKKQQQQQTTTSDCRQ    284
Takifugu        LLVPVDEEHHSHLILGVLLTLRYLMPLLQQQVNTISLKGSFGVMQKE----ADVQPAPEQ    298
Danio           LVVPVDEEHSSHLILGVLLTLRYLMPLIQQQTPSTSLKGSFGLVRKE----ADVTPAPEQ    298
Bos             LLVPVEGEHPTLLILGVLLALRYLVPLLQQQVKDTSLKGSFGVTRKE----MEVSPSTEQ    339
Homo            LLVPVEDEHSTLLILGVLLTLRYLVPLLQQQVKDTSLKGSFGVTRKE----MEVSPSAEQ    353
Rattus          LLVPMEEDHPTLLILGVLLTLRCLVPLLQQQVKDTSLKGSFGVTRKE----MEVSPSAEQ    332
Mus             LLVPMEEEHSTLLILGVLLTLRCLVPLLQQQVKDTSLKGSFGVTRKE----MEVSPSTEQ    332
                *::  :    :  :**.*  ** *:* : :     * :.* .:  *:      .    .*

Drosophila      IIEIYDYCLHLLSTQHTANHAIINATLEVINGILQAVDAASDGQCSQSLGQSLRQLLCNQ    344
Takifugu        LLQVYELTLHYTQH---WDHNVVTAALELLQQTLRTPPPE------------LLHVLITAG    344
Danio           LIQVYELTLHYTQH---WDHNVVTASLELLQQMFRTPPPE------------LLNVLITRG    344
Bos             LVQVYELTLHYTQH---QDHNVVTGALELLQQLLRTPPPE------------LLRVLTTAG    385
Homo            LVQVYELTLHHTQH---QDHNVVTGALELLQQLFRTPPPE------------LLQTLTAVG    399
Rattus          LVQVYELTLHHTQH---QDHNVVTGALELLQQLFRTPPPE------------LLQALTTPG    378
Mus             LVQVYELTLHHTQH---QDHNVVTGALELLQQLFRTPPPE------------LLQALTTPG    378
                ::::*:  **  .     :* ::..:**:::  :::                *. *

Drosophila      QLQHNEYLRRRKSLKNQIFQLKNYEVATSQHQLEDEDENEDVDELVVGATAMQM---KKN    401
Takifugu        SIQHASVF---------------------RQDIESRARSGSILELIAGGGSTCSPLLHRK    383
Danio           KITHTSVF---------------------REEMESRARSGSILELIAG-GSTCSPLLLRK    382
Bos             GVRQLAAS---------------------KDEPGGRSRSGSIVELIAGGGSSCSPVLSRK    424
Homo            GIGQLTAA---------------------KEESGGRSRSGSIVELIAGGGSSCSPVLSRK    438
Rattus          GLGQLTLV---------------------REEAGGRGRSGSIVELLAGGGSSCSPVLSRK    417
Mus             GLGQLTLV---------------------QEEARGRGRSGSIVELLAGGGSSCSPVLSRK    417
                 : :                         :.:  .. .. .: **:.*  :       ::

Drosophila      SNAKLQQAKCREQQQHQHQQQLEVDNSSLGINAGEDAPTEAPSS---VADEGGEPEST-K    457
Takifugu        HRGKMLSGEE-DALEDDPEKT-DVTTGYFTAVGADN---------------------SSA    420
Danio           QKGKLLSGEE-EGLEDDPER-AEVTTGSFTASVGGDSSSEAP-SSSGVSSL---------    430
Bos             QKGKVLFGEA-APLEDDSEPRSEGSSPAFSASVKGEVGGELAAS-SGISTPGSASSAADS    482
Homo            QKGKVLLGEE-EALEDDSESRSDVSSSALTASVKDEISGELAA-SSGVSTPG-------S    489
Rattus          QKGKVLLGEE-EALEDDSESRSDVSSSAFAASVKSEIGGELAASSSGVSTPG-------S    469
Mus             QKGKVLLGEE-EALEDDSESRSDVSSSAFAASVKSEIGGELAA-SSGVSTPG-------S    468
                 ..*:  .:     :.: :   :  .  :      :

Drosophila      LR-CHIRNAARSISECVASDEDKQGQGHRQQRDEDGVVVAEDDDDDDDDDDDDDMELLS    516
Takifugu        AQVDIITQQPRSSQHTIQPGDSVDLSASSEQGGRGGGASASD---TPESPNDEEDMLSRS    477
Danio           GTSDIITEQPRSSQHALQPGDSVDLSASEQGVGPD-----------TPDEEDEEDMLSRS    479
Bos             VGHDIITEQPRSQH-TLQ-TDAVDLAACDLTS--------------AATDGDEEDILSHS    526
Homo            AGHDIITEQPRSQH-TLQ-ADSVDLASCDLTS--------------SATDGDEEDILSHS    533
Rattus          VGHDIITEQPRSQH-TLQ-ADSVDLSGCDLTS--------------AATDGDEEDILSHS    513
Mus             VGHDIITEQPRSQH-TLQ-ADSVDLSGCDLTS--------------AATDGDEEDILSHS    512
                     * :  **    :   :  :  .                        *::*:   *

Drosophila      AECDDFTTLSQLNEQQQALSAALKLPTTTAASSGGAATSQDDKLIDVDADVGGLPKPQHQ    576
Takifugu        SSCGAN------------------ITPETVE----DAT--PENPAQEGRPVGGSGAYDHS    513
Danio           SSGGAG------------------LVSTSGD----LVT--DANQMS--------AGAVSS    507
Bos             SSQMSA------------------VPS----------D--PAMDLN--------DGTQAS    548
Homo            SSQVSA------------------VPS----------D--PAMDLN--------DGTQAS    555
Rattus          SSQFSA------------------VPS----------D--PAMDLN--------DGTQAS    535
Mus             SSQFSA------------------VPS----------D--PAMDLN--------DGTQAS    534
```

```
                :.                      :                    .             .

Drosophila      SS----LQNLLAGSDDKSQHLSDIDNESFNSIDFDAEITIAGSKEQQQQHPPADDSVESG     632
Takifugu        LPPSDSSQTTTEGPDSA------------VTPSDVAELVLDGSESQYSG-----------     550
Danio           SPPSESSQTTTEGPDSA------------VTPSDCAELVLDGSESQYSG-----------     544
Bos             SPISDSSQTTTEGPDSA------------VTPSDSSEIVLDGTDSQYPG-----------     585
Homo            SPISDSSQTTTEGPDSA------------VTPSDSSEIVLDGTDNQYLG-----------     592
Rattus          SPISDSSQTTTEGPDSA------------VTPSDSSEIVLDGADSQYLG-----------     572
Mus             SPISDSSQTTTEGPDSA------------VTPSDSSEIVLDGADSQYLG-----------     571
                       *.   * *.              : .  :*:.: *:..*

Drosophila      DATAIGTFFNNLLSHSNAASESVSKLFRQSSGSKSTPSKSASTPAPADKSDAISAASLTL     692
Takifugu        --MQIGTLQ-------------------------DEEDEGTATSSQEDPPDPFLRSAL--     581
Danio           --MQIGTLQ-------------------------DEEE-EGSAPPPDKPPEPFSQSAL--     574
Bos             --MQVGQTQ-------------------------DEDE-DATAVLPDEDAETFRNSSIDF     617
Homo            --LQIGQPQ-------------------------DEDE-EATGILPDEASEAFRNSSM--     622
Rattus          --VQIGQPQ-------------------------EEDEEEAAGVLSGEVSDVFRNSSL--     603
Mus             --MQIGQPQ-------------------------EDDEEGAAGVLSGEVSDVFRNSSL--     602
                    :*                            .  .   :     .  : :  :::

Drosophila      SLTSLASSNLEPPERQPLIAETPTPVEDSCSITASHTASTALMMDAPAVEVAASKPETPQ     752
Takifugu        ---ALSKPH--------LF-ES----RGHNRQGS-DS----------SVDRFIPKDE-PP     613
Danio           ---ALSKPH--------LL-EG----KGHNRQSS-DS----------SVDRFIPKEE-VL     606
Bos             E--ALQQAH--------LL-KS----MGHCRQSS-DS----------SVDKFVSREE-AA     650
Homo            ---ALQQAH--------LL-KN----MSHCRQPS-DS----------SVDKFVLRDE-AT     654
Rattus          ---ALQQAH--------LL-ER----MGHSRQPS-DS----------SVDKFVSKDE-VA     635
Mus             ---ALQQAH--------LL-ER----MGHSRQPS-DS----------SIDKYVTRDE-VA     634
                   :* . :        *: :      .     : .:          :::    : *

Drosophila      LRGTPNANPFLVENSPLRQTVVGRALITVKIGSIL--EQSLVYYTARLVAARFLLSGQAA     810
Takifugu        EP-EP-------DNKMSR--------IKGAIGHYTDRGAEPVVHCVRLLSASFLLTGQKN     657
Danio           EPAEL-------DNKPSR--------IKGDIGHYTDPKEEPLMHCVRLLAASFLLTGQRN     651
Bos             EPGDP-------ENKPCR--------VKGDIGQSTDEDSAPLVHCVRLLSASFLLTGEKN     695
Homo            EPGDQ-------ENKPCR--------IKGDIGQSTDDDSAPLVHCVRLLSASFLLTGGKN     699
Rattus          EAGDP-------ESKPCR--------IKGDIGQPNDDDSAPLVHCVRLLSASFLLTGEKK     680
Mus             EASDP-------ESKPCR--------IKGDIGQPNDDDSAPLVHCVRLLSASFLLTGEKK     679
                            :..  *        :.  **         : : .**::* ***:*

Drosophila      GLQPDSISRVSIKSLSLAVIAQCVRLAPKILQLSLEISEQELQLLEEATSQIGSGDSTQV     870
Takifugu        GLTPDRDVRVSVKALAVSCVGAAAALHPEAFFNSLYLEPLDG------------------     699
Danio           GLVPDNEVRVSVKALAVSCVGAAAALLPEAFFNLLYLQPLDG------------------     693
Bos             ALVPDRDVRVSVKALALSCIGAAVALHPESFFSKLYRAPLDT------------------     737
Homo            VLVPDRDVRVSVKALALSCVGAAVALHPESFFSKLYKVPLDT------------------     741
Rattus          ALVPDRDVRVSVKALALSCIGAAVALHPESFFSKLYKVPLST------------------     722
Mus             ALVPDRDVRVSVKALALSCIGAAVALHPESFFSRLYKVPLNT------------------     721
                 * **   ***:*:*::: :. .. * *: :   *     .

Drosophila      SSPQSSDNSQVGGEKPPLDSSLVPTSLEENLLLLDIKDDHFGPSTCPAYLQSATPTLSRS     930
Takifugu        ------------------------------------------------------------     699
Danio           ------------------------------------------------------------     693
Bos             ------------------------------------------------------------     737
Homo            ------------------------------------------------------------     741
```

```
Rattus          ------------------------------------------------------------   722
Mus             ------------------------------------------------------------   721

Drosophila      ADASVLLLEGGTTSSRSAKKSEEMLSKSEIIESSYRPTVAVEDVPPLSMPPRPPKRTKST   990
Takifugu        ------------------------------------------------------------   699
Danio           ------------------------------------------------------------   693
Bos             ------------------------------------------------------------   737
Homo            ------------------------------------------------------------   741
Rattus          ------------------------------------------------------------   722
Mus             ------------------------------------------------------------   721

Drosophila      RSRVGVLGTSSTTESSSPQSRQKLSDILLFHDHCDPILRGGVQQVVGNFLQSSGAGLFLD   1050
Takifugu        ---------------LRAEEQQYISDVLGFIDHGDPQIRGATAILCAAIIQAALSKMRYN   744
Danio           ---------------QQTEEQQYISDILQYIEHGDHQIRGATAILCGALIQAIQLKTRYN   738
Bos             ---------------VEYPEEQYVSDVLNYVDHGDPQVRGATAILCGTLICSVLGRCRFH   782
Homo            ---------------TEYPEEQYVSDILNYIDHGDPQVRGATAILCGTLICSILSRSRFH   786
Rattus          ---------------MESTEEQYVSDILNYIDHGDPQVRGATAILCGTLVYSILSRSRLR   767
Mus             ---------------TESTEEQYVSDILNYIDHGDPQVRGATAILCGTLVYSILSRSRLR   766
                                   ..* :**:* : :* *  :**..  : . :: :

Drosophila      LQRGLGLQHLLAILLKGFEDEIHTVVIQALNAFDKIFPNVVSKYLTEPPCHYHAHQQQQQ   1110
Takifugu        IHSWL--------------ASVQSK-----------------------------------   755
Danio           TETWL--------------SQIQSV-----------------------------------   749
Bos             VAGWM--------------GAVRAR-----------------------------------   793
Homo            VGDWM--------------GTIRTL-----------------------------------   797
Rattus          VGDWL--------------GTIRAL-----------------------------------   778
Mus             VGDWL--------------GNIRTL-----------------------------------   777
                    :                :::

Drosophila      QQKEQQQQEQDNQKLEQDLQRHSSGQQKRSGQAQTFGQQTFAKDQDNALSSQRQQQRRPN   1170
Takifugu        -----------------------------TGN--PLSLVDLVPLL--------QKALKDE   776
Danio           -----------------------------TGS--SVTLENFVPLL--------QRSLKDE   770
Bos             -----------------------------TGN--TFSLADCIPLL--------QKTLKDE   814
Homo            -----------------------------TGN--TFSLADCIPLL--------RKTLKDE   818
Rattus          -----------------------------TGN--TFSLVDCIPLL--------QKTLKDE   799
Mus             -----------------------------TGN--TFSLVDCIPLL--------QKTLKDE   798
                                             :*.   .                 ::  : :

Drosophila      DAGTCANSSATDNDELLAALLNDFQLQSTGMRQQPKNNSTDTGQSGNEPDLEPNPNAAVE   1230
Takifugu        SSVTCKMACSAVR-------------------------------------------HCIM   793
Danio           SSVTCKMACAAVR-------------------------------------------HCIM   787
Bos             SSVTCKLACAAVR-------------------------------------------LCVM   831
Homo            SSVTCKLACTAVR-------------------------------------------NCVM   835
Rattus          SSVTCKLACTAVR-------------------------------------------HCVL   816
Mus             SSVTCKLACTAVR-------------------------------------------HCVL   815
                .: **  :.:: .                                            .:

Drosophila      PFCVFAIS--PKLLLSKLRLCHHNKYWLVQNKYAEVISNLNYVLLRSYYANFRCAIDNKN   1288
Takifugu        SLCGSTLSELGLRLVVDLFALKDSSYWLVRTELLETLAEMDFRLVNFLERKS----EALH   849
Danio           ALCNGSLSELGLQLLIDLLTLKNCSYWLVRTELLETLAEIDFRLISFLERKT----EKLH   843
```

```
Bos            SLCSSSYSAWGLQLITNLLALRSSSYWLVRTELLETVAEIDFRLVSFLEAKA----ESLH    887
Homo           SLCSSSYSELGLQLIIDVLTLRNSSYWLVRTELLETLAEIDFRLVSFLEAKA----ENLH    891
Rattus         SLCSSSYSDLGLQLLIDMLPLKNSSYWLVRTELLETLAEIDFRLVSFLEAKA----ESLH    872
Mus            SLCSSSYSDLGLQLLIDMLPLKNSSYWLVRTELLDTLAEIDFRLVSFLEAKA----ESLH    871
                :*  : *     *: .:   :  .****:.:  :.:::::: *:      :     :  :

Drosophila     SGARKQDSKWPPMDASSVCHSVRDAEGEDIVCTYEAQFLAELLHLLGDDDARVREHAACC    1348
Takifugu       KGE----HHY-----------------TGRLRLQERVLNDVVIQLLGDDDPRVRHVAASA    888
Danio          KGE----HHY-----------------TGLLRLQDRVLNDVVLYLLGDEDPRVRHVAANT    882
Bos            RGA----HHY-----------------TGLLKLQERVLSNVVIHLLGDEDPRVRHVAAAS    926
Homo           RGA----HHY-----------------TGLLKLQERVLNNVVIHLLGDEDPRVRHVAAAS    930
Rattus         RGA----HHY-----------------TGFLKLQERVLNNVVIYLLGDEDPRVRHVAATT    911
Mus            RGA----HHY-----------------TGFLKLQERVLNNVVIYLLGDEDPRVRHVAATS    910
                *      ::                  . :   :  :   :: ****:* ***. **

Drosophila     LCRFIMQTARQDPSQDQGAGGGGGDDIEGNGNVNV-----ETQQ-TNFNLL---WDFFDY    1399
Takifugu       VSRLVSRLFFD---CDQGQADPVVAIARDQSSVYLQLLMHETQPPSQLTVSTITRTYRGF    945
Danio          IGRLVPRLFFD---CDQGQTDPVVAIARDQSSVHLQLLMHETQPPSQFTVSTITRTYRGF    939
Bos            LMRLVPKLFYK---CDQGQADPVVAVARDQSSVYLTLLMHETQPPSHFSVSAVTRIYRGY    983
Homo           LIRLVPKLFYK---CDQGQADPVVAVARDQSSVYLKLLMHETQPPSHFSVSTITRIYRGY    987
Rattus         LTRLVPKLFYK---CDQGQADPVVAVARDQSSVYLKLLMHETQPPSHFSVSTITRIYRGY    968
Mus            LTRLVPKLFYK---CDQGQADPVVAVARDQSSVYLKLLMHETQPPSHFSVSTITRIYRGY    967
               : *:: :    .    ***   .      ..:..* :      ***  :::.:        :  .:

Drosophila     RIFGSM-SVTLRNL-FRASSTIVPPLAELDALATSNSAPSYPDTGSTSGSSTSTSASSGG    1457
Takifugu       NLSNNVADVTVENNLSRVVTAVSHA------F----------------TSSTSRALTFGC    983
Danio          NLCQSAPDVTVENNLSRVITAISHA------L----------------TSSTSRAMTFGC    977
Bos            NLLPSITDVTLENNLSRVIAAVSHE------L----------------ITSTTRALTFGC    1021
Homo           NLLPSITDVTMENNLSRVIAAVSHE------L----------------ITSTTRALTFGC    1025
Rattus         SLLPSVTDVTMENNLSRVVAAVSHE------L----------------ITSTTRALTFGC    1006
Mus            SLLPSITDVTMENNLSRVVAAVSHE------L----------------ITSTTRALTFGC    1005
                :   .  .**:.*    *. :::          :                 :**: :  : *

Drosophila     SAAAVSAASAYFEASYGIGIA-------------------EGHVFALASASQRQIAQEEK    1498
Takifugu       CEALCLLAVHFPICTWTTGWHCGHISSQSSFSS--RVGRSRGRTLSVSQSGSTPASST--    1039
Danio          CEALCLLSSTFPVCNWSTGWHCGFVSSSVFHLNRSSQYRSRGRSFSLSQSGS--------    1029
Bos            CEALCLLSTAFPVCIWSLGWHCGVPPLS-----------------------A--------    1050
Homo           CEALCLLSTAFPVCIWSLGWHCGVPPLS-----------------------A--------    1054
Rattus         CEALCVLSAAFPVCTWSLGWHCGVPPLS-----------------------A--------    1035
Mus            CEALCLLSAAFPVCTWSLGWHCGVPPLS-----------------------A--------    1034
               . *    :  :  . :  *

Drosophila     VLAKVLYRLTNKLMTLNDKNVQFGIIYALRLLLRHFNFVDYQQVWLEFNFVE--------    1550
Takifugu       ---------TSSAVDPERRTLTVGTANMV--------LSLLSSAWFPLDLSAHQDALLLC    1082
Danio          -------------NEEVRRSLTVGVASMV--------LSLISSAWFPLDLSAHQAALLLA    1068
Bos            -------------SDESRKSCTVGMVSMI--------LTLLSSAWFPLDLSAHQDALILA    1089
Homo           -------------SDESRKSCTVGMATMI--------LTLLSSAWFPLDLSAHQDALILA    1093
Rattus         -------------SDESRKSCTVGMASMI--------LTLLSSAWFPLDLSAHQDALILA    1074
Mus            -------------SDESRKSCTVGMASMI--------LTLLSSAWFPLDLSAHQDALILA    1073
                                   :.   .*    :         :   ...*: :::

Drosophila     ---------ICISYAYYNNA--TAA---DLGCQNDLIDVMGKLMAGAMLSSGEPNTAHLD    1596
```

```
Takifugu        GNLLAAVAPKCLRNPWAGEDDSSSSSTNTSGGTHKMEEPWAAL-------SDRAFVAMVE    1135
Danio           GNLLAAVAPKCMKSPWAGEEESSPA-------SSKVEEPWPAL-------NDRSLVVMVE    1114
Bos             GNLLAASAPRSLRSSWASEDEASTA-------ATKQEEAWPAL-------GDRTLVPMVE    1135
Homo            GNLLAASAPKSLRSSWASEEEANPA-------ATKQEEVWPAL-------GDRALVPMVE    1139
Rattus          GNLLAASAPKSLRSSWASEEEGSSA-------ATRQEEIWPAL-------GDRTLVPMVE    1120
Mus             GNLLAASAPKSLRSSWTSEEEANSA-------ATRQEEIWPAL-------GDRTLVPLVE    1119
                          .:   : .:   . :            :    *       ...  .  ::

Drosophila      FLLRHSVKMLNIYYHLVTNQRPPTAGSQ--------------------------------    1624
Takifugu        QLFSHLLKVLNICAHVLDDTPPGPPVKATLPSLTNTPSLSPIRRKGKDKDAVDSSSAPLS    1195
Danio           QLFSHLLKILNICAHVLDDTPPGPAVKASLPSLANTPSLSPIRRKGKEKDMMEAGTTPMS    1174
Bos             QLFSHLLKVINICAHVLDDVAPGPAVKAALPSLTNPPSLSPIRRKGKEKEPGEQASVPVS    1195
Homo            QLFSHLLKVINICAHVLDDVAPGPAIKAALPSLTNPPSLSPIRRKGKEKEPGEQASVPLS    1199
Rattus          QLFSHLLKVINICAHVLDDVTPGPAIKAALPSLTNPPSLSPIRRKGKEKEPGEQTSTPMS    1180
Mus             QLFSHLLKVINICAHVLDDVTPGPAIKAALPSLTNPPSLSPIRRKGKEKEPGEQASTPMS    1179
                 *: * :*::**  *:: :  *    .

Drosophila      --SGSSSSKQPKSELFAREQPAATLQALGYFAGDYVYMKLYNILRGANDSYKITINQEAG    1682
Takifugu        PKKGNEANTG-RPTESTGSTAVHKSTTLGSFYHLPPYLKLYDVLKATHANFKVMLDLHSN    1254
Danio           PKKGGETNTG-RAADSTATAAVNKSTTLGSFYHLPPYLKLYDALRATHANYKVTLDLHNP    1233
Bos             PKKGSEASPASRPPETSGPVATNKSSSLGSFCHLPSYLKLHDVLKATHANYKVTLDLQSS    1255
Homo            PKKGSEASAASRQSDTSGPVTTSKSSSLGSFYHLPSYLKLHDVLKATHANYKVTLDLQNS    1259
Rattus          PKKGGEASTASRQSDTSGPVTASKSSSLGSFYHLPSYLRLHDVLKATHANYKVTLDLQNS    1240
Mus             PKKVGEASAASRQSDTSGPVTASKSSSLGSFYHLPSYLKLHDVLKATHANYKVTLDLQNS    1239
                  . ..:.   :    :    . .  :** *     *::*:: *:.:: .:*: :: .

Drosophila      SL-LICLLKTCLHAVSLCLEGMASASPPELKLIEEILHYLTRLINYAPAECVACLRQLLK    1741
Takifugu        QEKFGSFLRAALDVLSQLLELA--TLNDINKCVEEILGYLKSCFSREPTMATVCVQQLLK    1312
Danio           NEKFGGFLRSALDVLSQLLELA--TLHDIGKCVEEILGYLKSCFSREPTMATVCVQQLLK    1291
Bos             GEKFGGFLRSALDVLSQILELA--TLQDIGKCVEEILGYLKSCFSREPMMATVCVQQLLK    1313
Homo            TEKFGGFLRSALDVLSQILELA--TLQDIGKCVEEILGYLKSCFSREPMMATVCVQQLLK    1317
Rattus          TEKFGGFLRSALDVLSQILELA--TLQDIGKCVEEVLGYLKSCFSREPMMATVCVQQLLK    1298
Mus             TEKFGGFLRSALDVLSQILELA--TLQDIGKCVEEVLGYLKSCFSREPMMATVCVQQLLK    1297
                   :  :*::.*..:*  **    :     * :**:* **.  :.  *  ...*::****

Drosophila      YLFAQNYASQVRLQ---PSAIGGNGSEIG--------HHAAFMRPYFAAKGRGHGASSTL    1790
Takifugu        TLFGTNLASQYEGFLSGPSRSQGKALRLGSSSLRPGLYHYCFMAPYTHFTQALADA----    1368
Danio           ALFGTNLASQYEGASSNPCRSQGKALRLGSSSVRPGLYHYCFMAPYTHFTQALADA----    1347
Bos             TLFGTNLASQLDGLSSHASKSQGRAQRLGSSSARPGLYHYCFMAPYTLFTQALADA----    1369
Homo            TLFGTNLASQFDGLSSNPSKSQGRAQRLGSSSVRPGLYHYCFMAPYTHFTQALADA----    1373
Rattus          TLFGTNLASQFDGLSSNPSKSQCRAQRLGSSSVRPGLYHYCFMAPYTHFTQALADA----    1354
Mus             TLFGTNLASQFDGLSSNPSKSQCRAQRLGSSSVRPGLYHYCFMAPYTHFTQALADA----    1353
                 **. * ***        .    .. .:*        :* .** **   .    .*

Drosophila      LPTINSKPAVAVGSQRGAPTDARQPIDA-GP---LQDMGMLFVHGLQPP---TPPAGDCV    1843
Takifugu        -------------SLRNMVQ-AEHEQDTSGWFDVMQKTSNQLRSNIANAARHRGDKNAIH    1414
Danio           -------------SLRNMVQ-AEQEQDASGWFDVMQKVSNQLRSSITNVTRHRGDKNAIH    1393
Bos             -------------SLRNAAQ-AEQDQDTAGWFDVLQKVSTQLKTNLTSVTKNRADKNAIH    1415
Homo            -------------SLRNMVQ-AEQENDTSGWFDVLQKVSTQLKTNLTSVTKNRADKNAIH    1419
Rattus          -------------SLRNMVQ-ADQEHDASGWFDVLQKVSAQLKTNLTSVTKNRADKNAIH    1400
Mus             -------------SLRNMVQ-AEQERDASGWFDVLQKVSAQLKTNLTSVTKNRADKNAIH    1399
                             * *.    * :  *: *    :*. .  :  .:          .
```

```
Drosophila      RLIKLFEPMVIYCLTLFMKSNA-LVQAPILRLLSQLLDLNVTYSILDSKNVIFDQVLSNM    1902
Takifugu        NHIRLFEPLVIKALKQYTTSTSVALQRQVLDLLAQLVQLRVNYCLLDSDQVFIGFVLKQF    1474
Danio           NHIRLFEPLVIKALKQYTTSTSVALQRQVLDLLAQLVQLRVNYCLLDSDQVFIGFVLKQF    1453
Bos             NHIRLFEPLVIKALKQYTTTTSVQLQKQVLDLLAQLVQLRVNYCLLDSDQVFIGFVLKQF    1475
Homo            NHIRLFEPLVIKALKQYTTTTCVQLQKQVLDLLAQLVQLRVNYCLLDSDQVFIGFVLKQF    1479
Rattus          NHIRLFEPLVIKALKQYTTTTSVQLQKQVLDLLAQLVQLRVNYCLLDSDQVFIGFVLKQF    1460
Mus             NHIRLFEPLVIKALKQYTTTTSVQLQKQVLDLLAQLVQLRVNYCLLDSDQVFIGFVLKQF    1459
                . *:****:** .*. : .:..  :*  :* **:**::*.*.*.:***.:*::. **.::

Drosophila      DLIEGGIDRNAFIMVPPMLRFLVQLT--HKSDRQLITIPKIISITNNLLANGSVRVVA-L    1959
Takifugu        EYIEVGQFRDSEAIIPNIFFFLVLLSYERYHSKQIISIPKIIQLCDGIMASGRKAVTHAI    1534
Danio           EYIEVGQFRDSEEIVPNIFFFLVLLSYERYHSKQIISIPKIIQLCDGIMASGRKAVTHAI    1513
Bos             EYIEVGQFRESEAIIPNIFFFLVLLSYERYHSKQIIGIPKIIQLCDGIMASGRKAVTHAI    1535
Homo            EYIEVGQFRESEAIIPNIFFFLVLLSYERYHSKQIIGIPKIIQLCDGIMASGRKAVTHAI    1539
Rattus          EYIEVGQFRESEAIIPNIFFFLVLLSYERYHSKQIIGIPKIIQLCDGIMASGRKAVTHAI    1520
Mus             EYIEVGQFRESEAIIPNIFFFLVLLSYERYHSKQIIGIPKIIQLCDGIMASGRKAVTHAI    1519
                : ** *  *::  ::* :: *** *:  :  .:*:* *****.: :.::*.*   *.  :

Drosophila      LALKTLSYELFFMHSQLEEALDTEGHNSGRDACQSPLSAAPTPETREALLAQRRELDTQR    2019
Takifugu        PALQPIVHDLFVLRGSN-------------------------------KADAGKELETQK    1563
Danio           PALQPIVHDLFVLRGSN-------------------------------KADAGKELETQK    1542
Bos             PALQPIVHDLFVLRGTN-------------------------------KADAGKELETQK    1564
Homo            PALQPIVHDLFVLRGTN-------------------------------KADAGKELETQK    1568
Rattus          PALQPIVHDLFVLRGTN-------------------------------KADAGKELETQK    1549
Mus             PALQPIVHDLFVLRGTN-------------------------------KADAGKELETQK    1548
                 **: : ::**.::.                                      :**:**:

Drosophila      EVVLGMLEKFIEARPSQQVLALLLLFERSVQQLDTPPYR--SAQDADAVYGTLCRGLCSR    2077
Takifugu        EVVVSMLLRLVQYHQV---LEMFILVLQQCHKENEDKWKRLSRQIADVILP----MIAKQ    1616
Danio           EVVVSMLLRLIQHHQV---LEMFILVLQQCHKENEDKWKRLSRQVADIILP----MIGKQ    1595
Bos             EVVVSMLLRLIQYHQV---LEMFTLVLRQCHKESEDRWKRLSRQVADVILP----MLAKQ    1617
Homo            EVVVSMLLRLIQYHQV---LEMFILVLQQCHKENEDKWKRLSRQIADIILP----MLAKQ    1621
Rattus          EVVVSMLLRLIQYHQV---LEMFILVLQQCHKENEDKWKRLSRQVADIILP----MLAKQ    1602
Mus             EVVVSMLLRLIQYHQV---LEMFILVLQQCHKENEDKWKRLSRQVADIILP----MLAKQ    1601
                ***:.** :::: :     * :: *. :. :: .   ::  * * ** :       : .:

Drosophila      QWRLHNAGDLRLLESCFRNNGNHVLADSKRFLQLLQLFIEQG-VGNF--------GDLAL    2128
Takifugu        QMHLDSPEALGVLNTLFETVAPSSLRPVDMLL--KSMFTTPVTMASVATVQLWVSGILAV    1674
Danio           QMHLDSHEALGVLNTLFESVAPSSLRPVDMLL--KSMFITPSTLASVGTVQLWVSGILAI    1653
Bos             QMHIDSHEALGVLNTLFEILAPSSLRPVDMLL--RSMFVTPDTLAAVSTVQLWVSGILAI    1675
Homo            QMHIDSHEALGVLNTLFEILAPSSLRPVDMLL--RSMFVTPNTMASVSTVQLWISGILAI    1679
Rattus          QMHIDSHEALGVLNTLFEILAPSSLRPVDMLL--RSMFITPSTMASVSTVQLWISGILAI    1660
Mus             QMHIDSHEALGVLNTLFEILAPSSLRPVDMLL--RSMFITPSTMASVSTVQLWISGILAI    1659
                * ::..   * :*:: *.  .   *   . :*   .:*     :. .        * **:

Drosophila      AMVMLSNVILKTEEIYLVNHI----KLYLKNNPTAERRLQALMPSSPSAAPHWQDEAPST    2184
Takifugu        LRVLVS---QSTEDIVLSRIHELSLSPHLLSCHTIKRLQQPNLSPSDQPAGD--------    1723
Danio           LRVLIS---QSTEDIILSRIQELSLSPYLLSCPTIRRLCDDDVPAPPE--TL--------    1700
Bos             LRVLIS---QSTEDIVLSRIQELSFSPYLVSCPTISRLRDGDSTSAL-------------    1719
Homo            LRVLIS---QSTEDIVLSRIQELSFSPYLISCTVINRLRDGDSTSTL-------------    1723
Rattus          LRVLIS---QSTEDIVLSRIQELSFSPYLISCPVINRLRDGDSNPTL-------------    1704
```

```
Mus             LRVLIS---QSTEDIVLCRIQELSFSPHLLSCPVINRLRGGGGNVTL-------------    1703
                  *::*    .**:* * .      . :* .  .  *

Drosophila      SSAAAAARAAAASFSAGRSS-ISEINYFAKVLCEKLLACLE----VLLGLEP-SSSSHAY    2238
Takifugu        --------GQQNQEPNGEAQKSLPEETFARFLIQLVGVLLDDISSRHVKVDITEQQHTFY    1775
Danio           --------PVAMEDANGETTRFPPEETFARFLLQLVGVLLDDIANKQVKVDMSEQQHTFY    1752
Bos             -----------EEHTEGRQMKNLPEETFARFLLQLVGVLLEDIVTKQLRVEVSEQQQTFY    1768
Homo            -----------EEHSEGKQIKNLPEETFSRFLLQLVGILLEDIVTKQLKVEMSEQQHTFY    1772
Rattus          -----------GERSEGKQVKNLPEDTFSRFLLQLVGILLEDIVTKQLKVDMSEQQHTFY    1753
Mus             -----------GECSEGKQ-KSLPEDTFSRFLLQLVGILLEDIVTKQLKVDMSEQQHTFY    1751
                            .   *.       : *::.* : :   *:      : ::  ...   *

Drosophila      CQLTGRFMDALLNVCCRSRHKDALQSVFRLVLAESE---FLCKYYSLLLMSAAGLVGSY-    2294
Takifugu        CQQLGTLLMCLIHVFKSGMFRRITVAASRLLKGESGSGHSGIEFYPLEGLNS--MVHCLI    1833
Danio           CQQLGTLLMCLIHIFKSGMFRRITAAGSKLLKAEGGE---GGDFYTLEGLNS--LVLQLI    1807
Bos             CQELGTLLMCLIHIFKSGTFRRITAAASRLLR-DG-A---DGGFYGLESLNA--WVRSMV    1821
Homo            CQELGTLLMCLIHIFKSGMFRRITAAATRLFRSDG-C---GGSFYTLDSLNL--RARSMI    1826
Rattus          CQELGTLLMCLIHIFKSGMFRRITAAATRLFTSDG-C---EGSFYTLDSLNA--RVRAMV    1807
Mus             CQELGTLLMCLIHIFKSGMFRRITAAATRLFTSDG-C---EGSFYTLESLNA--RVRSMV    1805
                **  * :: .*:::   . .:    :  :*.  :.        :* *  :.    .

Drosophila      --LLDAVLLACLRLVLAMRLEEPVALLEQAAQLPLKTNLQ--------------------    2332
Takifugu        TTHPSLVLLWCQVLLIIDYTN--YSWWTEVHQTPKGHSLSCTKLLSPHSSGEGEEKP-ET    1890
Danio           TTHPSLVLLWCQVLLIINYTN--YTWWSEVHQTPRRHSLSSTKLLSPHSSGEEERPE--G    1863
Bos             PTHPALVLLWCQILLLVSHTD--YRWWAEVQQTPKRRSLSSTKSLSPETSGEDEDPDVAS    1879
Homo            TTHPALVLLWCQILLLVNHTD--YRWWAEVQQTPKRHSLSSTKLLSPQMSGEEEDSDLAA    1884
Rattus          PTHPALVLLWCQILLLINHTD--HRWWAEVQQTPKRHSLSCTKSLNPQISA-EEDSGSAA    1864
Mus             PTHPALVLLWCQILLLINHTD--HRWWAEVQQTPKRHSLSCTKSLNPQKSGEEEDSGSAA    1863
                      *** *  *::    :       :. * *   .*.

Drosophila      --RALLREVCRASA---GCDWSAQQVRRLFEGRYLNFLIADHLEFICELCQERPECGSLL    2387
Takifugu        RLAMINREIVRRGALILFCDYVC---QNLHDSEHLTWLIVNHVRDLIDLSHEPPVQDFIS    1947
Danio           KLTMCNREIVRRGALILFCDYVC---QNLHDSEHLTWLTVNHVSDLISLSHEPPVQDLIS    1920
Bos             KLGMCNREIVRRGALILFCDYVC---QNLHDSEHLTWLIVNHIQDLINLSHEPPVQDFIS    1936
Homo            KLGMCNREIVRRGALILFCDYVC---QNLHDSEHLTWLIVNHIQDLISLSHEPPVQDFIS    1941
Rattus          QLGMCNREIVRRGALILFCDYVC---QNLHDSEHLTWLIVNHIQDLISLSHEPPVQDFIS    1921
Mus             QLGMCNREIVRRGALILFCDYVC---QNLHDSEHLTWLIVNHIQDLISLSHEPPVQDFIS    1920
                      **: * .*    **: .   :.*.:..:*.:* .:*:  : .*.:* *  . :

Drosophila      QVALFRNAHRLSRQSVRIVLRLLGRLCE----PAERE------------ASGDVGSD-AD    2430
Takifugu        AV------HRNSAAS-GLFIQAIQSRCDNLNSPTMLKKTLQCLEGIHLSQSGSLLMLYVD    2000
Danio           AV------HRNSAAS-GLFIQAIQSRCDNLCTPVMLKKTLQCLEGIHLSQSGALLMLYVD    1973
Bos             AI------HRNSAAS-GLFIQAIQSRCENLSTPTMLKRTLQCLEGIHLSQSGAVLMLYVD    1989
Homo            AV------HRNSAAS-GLFIQAIQSRCENLSTPTMLKKTLQCLEGIHLSQSGAVLTLYVD    1994
Rattus          AI------HRNSAAS-GLFIQAIQSRCENLSTPTTLKKTLQCLEGIHLSQSGAVLTLYVD    1974
Mus             AI------HRNSAAS-GLFIQAIQSRCENLSTPTTLKKTLQCLEGIHLSQSGAVLTLYVD    1973
                 :      ** *  *  :.:: :   *:    *.  :             ** :    .*

Drosophila      PT-LQAMQLLSRLHQLYE------------GERSLQLPIERMARRLS--AQSGQPARNVI    2475
Takifugu        KLLSTPFRVLARMVDTLACRRVEMLLAETLQNSVAQLPLEELHRIQEYLQTSGLAQRHQR    2060
Danio           KLLNTPFRVLARMVDTLACRRVEMLLAETLQNSIAQLPLEELDRIQQYLQTSGLAQRHQR    2033
Bos             KLLCTPFRVLARMVDTLACRRVEMLLAANLQSSTAQLPAEELIRIQEHLQSSGLAQRHQR    2049
```

```
Homo            RLLCTPFRVLARMVDILACRRVEMLLAANLQSSMAQLPMEELNRIQEYLQSSGLAQRHQR    2054
Rattus          RLLGTPFRALARMVDTLACRRVEMLLAANLQSSMAQLPEEELNRIQEHLQNTGLAQRHQR    2034
Mus             RLLGTPFRALARMVDTLACRRVEMLLAANLQSSMAQLPEEELNRIQEHLQNSGLAQRHQR    2033
                      :: *:*: :               .   *** *.: *  .    :*   *:

Drosophila      YERLVEG-DLAGGEDQDALRTLLLKDLECRQDNETATPSRIIDESWLFAQLIKFATQHAD    2534
Takifugu        FYSLLDRFRATV-SDTSSP-STPVTSHPLDGDPPPAPELVIADKEWYVALVKSQCCLHGD    2118
Danio           FFSLLDRFRATVAEDTTSP-VAPITTHPLDGDPPPPPENVEPNKEWYVTLVKSQCCLRGE    2092
Bos             LYSLLDRLRLATAPGSHGP-TPPVTSHPLDGDGPLALEAVNPDKDWYVQLVKAQCWTRSD    2108
Homo            LYSLLDRFRLSTMQDSLSP-SPPVSSHPLDGDGHVSLETVSPDKDWYVHLVKSQCWTRSD    2113
Rattus          LYSLLDRFRLSTVQDSLSP-LPPVTSHPLDGDGHTSLETVNPDKDWYLQLVRSQCWTRSD    2093
Mus             LYSLLDRFRLSTVQDSLSP-LPPVTSHPLDGDGHTSLETVSPDKDWYLQLVRSQCWTRSD    2092
                   *::    :   .  .     :.      *          ::.* .  :   .  :.:

Drosophila      APQQQKQLMLL---------LLEIQSEPKLQRL-------LRSLGTEHEAKLLRHAIAGSL    2579
Takifugu        VSLLETTELLTKLPPADLLSVMSCKEFNLSLLCPCLSLGVQRLLRGQGSLLLETALQVTL    2178
Danio           GALYETTELLTKLPQSDLNAIMTCKDFNLCLLASCLSVGVQRVCKDHGAVLFDTAQQVTF    2152
Bos             SALLEGAELVSRIPAGDLGAFMMHSEFNLSLLAPCLGLGMREISGGQESPLFEAARTATL    2168
Homo            SALLEGAELVNRIPAEDMNAFMMNSEFNLSLLAPCLSLGMSEISGGQKSALFEAAREVTL    2173
Rattus          SALLEGAELVNRIPAEDMSDFMMSSEFNLSLLAPCLSLGMSEIANGQKSPLFEAARRVTL    2153
Mus             SALLEGAELVNRIPAEDMNDFMMSSEFNLSLLAPCLSLGMSEIANGQKSPLFEAARGVIL    2152
                    :   ::          .   .: :*  *       :  :   : : *:  *    :

Drosophila      AAMMSAFRQKCIQHAPHINYMQPTPLARVSCALLMSRVASTEATKCRNPPTGEQLDVARA    2639
Takifugu        EQLAGATGLL---PVPHHSFIPTS---H--PQSHWK------------------------    2206
Danio           ERLAGVIELL---PSPHQPLLPSS---KV-CPDYWQ------------------------    2181
Bos             DRVTVVVQQL---PAVHEAFQPFL---PTQPSAYWS------------------------    2198
Homo            ARVSGTVQQL---PAVHHVFQPEL---PAEPAAYWS------------------------    2203
Rattus          DRVTNVVQQL---PAVHQVFQPFL---PTEPTAYWS------------------------    2183
Mus             NRVTSVVQQL---PAVHQVFQPFL---PIEPTAYWN------------------------    2182
                  :  .          *                  .

Drosophila      VGALMACIRNAEQTALIYIDARLME----------KF--VVEHLLRREHLPQ--------    2679
Takifugu        ------------QLAEVYGDPGFYSRVLSLCRALSQYLLTVKQLPSSLRIPSDKEHLITT    2254
Danio           ------------KLNQVYGEPGFYQTVLSLCGVLSQYLLSLSKLPSSMHIPKDRETLITT    2229
Bos             ------------KLDDLFGDAALYRTLTTLAQALAQYLLVFSKLPSHLHLPPEKERDTVK    2246
Homo            ------------KLNDLFGDAALYQSLPTLARALAQYLVVVSKLPSHLHLPPEKEKDIVK    2251
Rattus          ------------KLNDLFGDTTSYQSLTTLARALAQYLVVLSKVPAPLHLPPEKEGHTVK    2231
Mus             ------------KLNDLLGDTTSYQSLTILARALAQYLVVLSKVPAHLHLPPEKEGDTVK    2230
                            :   :  :               ::   ..::    ::*

Drosophila      ----LLAYLGWLAGAAKQILAMPTRQESEQDALGVLLATVNTLLQQPRVWRELNASSDPS    2735
Takifugu        FTCAATEVVVWHLLQDQL---------PLSVDLQWALSCLCLALQQPCVWNKLSTPEYNT    2305
Danio           FSTLAIEVVVWRLLQDQL---------PLSVDMQMSLSCLCLALQQPAVWTHFTSHSYLT    2280
Bos             FMVMTVEALSWHLIHSRV---------PLSLDLQAGLDCCCLALQLPGLWSLLAAPDMVT    2297
Homo            FVVATLEALSWHLIHEQI---------PLSLDLQAGLDCCCLALQLPGLWSVVSSTEFVT    2302
Rattus          FVVMTLEALSWHLIHEQI---------PLSLDLQAGLDCCCLALQVPGLWGVLSSPEYVT    2282
Mus             FVVMTVEALSWHLIHEQI---------PLSLDLQAGLDCCCLALQVPGLWGVLSSPEYVT    2281
                        : *     :            .  :   *      ** * :*  . : .  :

Drosophila      LRCELLDLLDSVARCILQ--DTIFYRRHRRDRN-K----------AKGPAPQAIFL----    2778
Takifugu        HTCSLIYCLHHIILAVAVSPGDQLLHPERKKTKALRHSDDEDQVDSVHDNHTLEWQACEI    2365
```

```
Danio           HTCSIIHCIQLLIHAVAVGPGDQFLRPERKISEQS-----EDQVDSSD--NQREWHCCEI    2333
Bos             HACSLIHCVRFILEAIVVQPGDQLLSPERRTSTPKAAR--EDSVDSDTQNPQYITAACGM    2355
Homo            HACSLIYCVHFILEAVAVQPGEQLLSPERRTNTPKAISEEEEEVDPNTQNPKYITAACEM    2362
Rattus          HTCSLIHCVRFILEAIAVQPGDQLLGPESRSHTPRAVRKE--EVDSDIQNLSHITSACEM    2340
Mus             HACSLIHCVRFILEAIAVQPGDQLLGPESRSHTPRAVRKE--EVDSDIQNLSHVTSACEM    2339
                  *.::  :  :  .:    .  :   . :

Drosophila      -AKLIETQIEIESLASGRVLAGVEEARLQFAGQDLARFQVALSLVTSIGISLLRTHQFYA    2837
Takifugu        MAELVEGLQSVLSLGH------HR--------NTAFPAF--LTPTLRNIIISLSRLPLVNS    2410
Danio           MAELVEGLQTVLTLGH------HK--------NKNIPAF--LTPTLRNVIISLARLPLVNS    2378
Bos             VAEMVECLPSVLALGH------KR--------NSRTPAF--LTPVLRNIVISLARLPLVNS    2400
Homo            VAEMVESLQSVLALGH------KR--------NSGVPAF--LTPLLRNIIISLARLPLVNS    2407
Rattus          VADMVESLQSVLALGH------KR--------NSTLPSF--LTAVLKNIVVSLARLPLVNS    2385
Mus             VADMVESLQSVLALGH------KR--------NSTLPSF--LTAVLKNIVISLARLPLVNS    2384
                 *.::*    : :*.        .       .     *      : .:  :** *  . :

Drosophila      YAVTPHELIQQPGDQQQEQQADGKLPSIPVDSLSDVDVLRQFVKRLSIFGFTTRQQFEEY    2897
Takifugu        HTRVPPLVWKLGWSPQPGGEFGTTLPEIPVDFLQEKDVFREFLYRINTLGWSNRTQFEET    2470
Danio           YTRIPPLVWKLGWSPKLSGEFGTALPEIPVEFLQEKDVFREFLYRINTLGWSSRTQFEET    2438
Bos             YMRVPPLVWKLGWSPKPGGDFGTVFPEIPVEFLQEKEVFREFIYRINTLGWTSRTQFEET    2460
Homo            YTRVPPLVWKLGWSPKPGGDFGTAFPEIPVEFLQEKEVFKEFIYRINTLGWTSRTQFEET    2467
Rattus          YTRVPPLVWKLGWSPKPGGDFGTVFPEIPVEFLQEKEVLKEFIYRINTLGWTSRTQFEET    2445
Mus             YTRVPPLVWKLGWSPKPGGDFGTVFPEIPVEFLQEKEILKEFIYRINTLGWTNRTQFEET    2444
                :   *  : :   . :   : .  :*.***: *.: :::::*: *:. :*::.* ****

Drosophila      FMTCLLLINKL--Y-----DEHMVDQQEQFQIKQVCLQAILELLMTYKTFPIVGLANGQF    2950
Takifugu        WATLLGVLVTQPITMDQEEETQQEEDLERTQLNVLAVQAITSLVLSAMTLPTAGNPAVSC    2530
Danio           WATLLGVLVTQPITMDQEEETQQEEDLERTQINVLAVQAITSLVLSAMTLPTAGNPAVSC    2498
Bos             WATLLGVLVTQPLMMEQEES-PPEEDVERTQIHVLAVQAITSLVLSAMTVPVAGNPAVSC    2519
Homo            WATLLGVLVTQPLVMEQEES-PPEEDTERTQINVLAVQAITSLVLSAMTVPVAGNPAVSC    2526
Rattus          WATLLGVLVTQPLVMEQEES-PPEEDTERTQIHVLAVQAITSLVLSAMAVPVAGNPAVSC    2504
Mus             WATLLGVLVTQPLVMEQEES-PPEEDTERTQIHVLAVQAITSLVLSAMTVPVAGNPAVSC    2503
                : * * :: .         .    :: *: *:: :.:*** .*:::  :.* .*    .

Drosophila      HHT-----------TRWQRITCDSISLKKLHKVQLLVDAC--------------------    2979
Takifugu        LEQQPRNKSLKALETRFGRKLAV-IRGEVEREIQALVSKRDNVHTYHPYHAWDPVPSLSA    2589
Danio           PEQQPRNKILKALDTRFGRKLSV-IRGMVEREIQAMVSKRDNIATHFPYQAWDPVPSLSS    2557
Bos             LEQQPRNKPLKALDTRFGRKLSI-IRGIVEQEIQAMVSRRENAATHHLYQAWDPVPSLAP    2578
Homo            LEQQPRNKPLKALDTRFGRKLSI-IRGIVEQEIQAMVSKRENIATHHLYQAWDPVPSLSP    2585
Rattus          LEQQPRNKPLKALDTRFGRKLSM-IRGIVEQEIQEMVSQRENTATHHSHQAWDPVPSLLP    2563
Mus             LEQQPRNKPLKALDTRFGRKLSM-IRGIVEQEIQEMVSQRENTATHHSHQAWDPVPSLLP    2562
                 .            **: *  .  *     :::* :*.

Drosophila      ----NVFYQPNLERQLAYDNVIGTRTFAPNQYDLNFSWAQMEDQAAAGVGVGVGVGLSGG    3035
Takifugu        ASPGTLISHEKLLLQINTERELGNMDYKLGQVSIHSVWLGNNITPLREE-------EWGE    2642
Danio           STAGTLISHEKLLLQINTEREMGNMDYKLGQVSIHSVWLGNNITPLREE-------EWGE    2610
Bos             ATTGALISHDKLLLQLNPERELGDMSYKLGQVSIHSVWLGNSITPLREE-------EWDE    2631
Homo            ATTGALISHEKLLLQINPERELGSMSYKLGQVSIHSVWLGNSITPLREE-------EWDE    2638
Rattus          ATTGALISHDKLLLQINSEREPGNMSYKLGQVSIHSVWLGNNITPLREE-------EWDE    2616
Mus             ATTGALISHDKLLLQINPEREPGNMSYKLGQVSIHSVWLGNNITPLREE-------EWDE    2615
                     :: : :*  *:  :.  *   :  .* .::  *   .                .
```

```
Drosophila      EQANTADIKQSCDADVPDMAMRNYRHFTQLSGIDFRSSTQLVFDVLQQMIELNH------    3089
Takifugu        DEDDEADPPAP---TSPPLS--PINSRKHRAGVDIHSCSQFLLELYSQWVIPGSPSNRKT    2697
Danio           DEEDEADAPAP---ASPQLS--PINSRKHRAGVDIHSCSQFLLELYSQWILPGSPSSRKT    2665
Bos             EEE-ETEAPAP---SSPPTS--PINSRKHRAGVDIHSCSQFLLELYSRWILPSS-SARRT    2684
Homo            EEEEEADAPAP---SSPPTS--PVNSRKHRAGVDIHSCSQFLLELYSRWILPSS-SARRT    2692
Rattus          EEEEEADAPAP---TSPPVS--PVNSRKHRAGVDIHSCSQFLLELYSRWILPSS-AARRT    2670
Mus             EEEEESDVPAP---TSPPVS--PVNSRKHRAGVDIHSCSQFLLELYSRWILPSS-AARRT    2669
                ::   ::         *  :    .  .: :*:*::*.:*::::: .: :  .

Drosophila      -I----LVLPNLVKFCEICESRDHIKWIKERCLKLQEQVAMDDTISHQHIIYLLCRSQAL    3144
Takifugu        PTILISEVVRSLLAVSDLFTERNQFDMMFSTLMELQKLHPPEDEILNQYLVPAICKAAAV    2757
Danio           PVVLISEVVRSLLAVSDLFTERNQFDMMFSTLTELQKVHPPEDEILNQYLVPAICKAAAV    2725
Bos             PVTLISEVVRSLLVVSDLFTERSQFETMYLTLTELRKVHPSEDEILLQYLVPATCKAAAV    2744
Homo            PAILISEVVRSLLVVSDLFTERNQFELMYVTLTELRRVHPSEDEILAQYLVPATCKAAAV    2752
Rattus          PVILISEVVRSLLVVSDLFTERTQFEMMYLTLTELRRVHPSEDEILIQYLVPATCKAAAV    2730
Mus             PVILISEVVRSLLVVSDLFTERTQFEMMYLTLTELRRVHPSEDEILIQYLVPATCKAAAV    2729
                       *: .*: ..::  .* ::. :     :*:.    :* *  *:::   *:: *:

Drosophila      LIPSLGELQVLCSLIGNVYLKSTHSFIRIATLQGLLCLLECCSKTNTTMGRLSEELALLR    3204
Takifugu        LGMDKAIAEPVCRLLE-TTLRSTHLPSRMGALHGVLYVLECDLLDDTAKQLIPTVSEYLL    2816
Danio           LGMDKAIAEPVCRLLE-STLRSTHLPSRIGALHGVLYVLECDLLDDTARQLIPTVSEYLL    2784
Bos             LGMDKAVAEPVSRLLE-SALRSSHLPSTMGALHGVLYVLECDLLDDTAKQLVPVVTDYLL    2803
Homo            LGMDKAVAEPVSRLLE-STLRSSHLPSRVGALHGVLYVLECDLLDDTAKQLIPVISDYLL    2811
Rattus          LGMDKTVAEPVSRLLE-STLRSTHLPSQIGALHGILYVLECDLLDDTVKQLIPVVSDYLL    2789
Mus             LGMDKTVAEPVSRLLE-STLRSSHLPSQIGALHGILYVLECDLLDDTAKQLIPVVSDYLL    2788
                *  .    : :. *:    *:*:*    :.:*:*:* :***    :*.   :      *

Drosophila      SLIVGYINRHGIIDESPLPFSVEHTKLVWTL------NYSL---IEWTSKFVPQCHLLSN    3255
Takifugu        SNLRAIAHCVNL-------HNQQHVLVMCAVAFYMMENYPLDVGTEFMAGIIQLCGVMVS    2869
Danio           SNLRAIAHCVNL-------HNQQHVLVMCAVAFYMMENYPLDVGSEFNAGIIQLCCMILS    2837
Bos             SNLQGRAHCVSV-------HSQQHVLVMCATAFYLIENYPLDVGPDFSASVIQMCGVMLS    2856
Homo            SNLKGIAHCVNI-------HSQQHVLVMCATAFYLIENYPLDVGPEFSASIIQMCGVMLS    2864
Rattus          SNLKGIAHCVNI-------HSQQHVLVMCATAFYLMENYPLDVGPEFSASVIQMCGVMLS    2842
Mus             SNLKGIAHCVNI-------HSQQHVLVMCATAFYLMENYPLDVGPEFSASVIQMCGVMLS    2841
                * : .  :  .:       .. :*. :: :       ** *    :: : .:  * :: .

Drosophila      TIIAANNFLKTTADEELYLCVLHGLERMVVNSGVPPPGIQPTGKDAAAGEPGAEGSKAGV    3315
Takifugu        ----A---SEDSTPSIIYHCVLRGLERLLLSEQLSRVDG---------------------    2901
Danio           ----A---SEEATPSIIYHCVLRGLERLLLSEQLSRMDA---------------------    2869
Bos             ----G---SEEATPSVVYHCVLRGLERLLLSEQLSRLDA---------------------    2888
Homo            ----G---SEESTPSIIYHCALRGLERLLLSEQLSRLDA---------------------    2896
Rattus          ----G---SEESTPSIIYHCALRGLERLLLSEQLSRLDT---------------------    2874
Mus             ----G---SEESTPSIIYHCALRGLERLLLSEQLSRLDT---------------------    2873
                    .    : :: . :* *.*:****:::.. :   .

Drosophila      GVVVTPQMRHKIEKLALELLKMENEKFSIPALKLLLSCMYVGSAAQLENTELSNGIVQ--    3373
Takifugu        ---------EALVKLSVDRVNMPSPHRAMAALGLMLTCMYTG------KEKASPAARSAH    2946
Danio           ---------ETLVKLSVDRVNMPSPHRAMAALGLMLTCMYTGVAGEEGKEKGSP-GRPAD    2919
Bos             ---------ESLVKLSVDRVNVHSPHRAMAALGLMLTCMYTG------KEKVSP-GRASE    2932
Homo            ---------ESLVKLSVDRVNVHSPHRAMAALGLMLTCMYTG------KEKVSP-GRTSD    2940
Rattus          ---------ESLVKLSVDRVNVQSPHRAMAALGLMLTCMYTG------KEKASP-GRASD    2918
Mus             ---------ESLVKLSVDRVNVQSPHRAMAALGLMLTCMYTG------KEKASP-GRASD    2917
```

```
                         . : **::: ::: . : :: ** *:*:***.*      : : *

Drosophila      -----DDPEIIAQQNDKVDILLHCIKSSTRDAAWIYGQVLCQIIRDLVPPNEILTKVIKE    3428
Takifugu        SDPQVPDSESIIVAMERVSVLFDRIRKGLPSEARVVARILPQFLDDFFPPQDIMNKVIGE    3006
Danio           ADPTAPDSESVIVAMERVSVLFDRIRKGFPSEARVVARILPQFLDDLFPLQDVMNKVIGE    2979
Bos             PSTAAPDSESVIVAMERVSVLFDRIRKGFPCEARVVARVLPQFLDDFFPPQDVMNKVIGE    2992
Homo            PNPAAPDSESVIVAMERVSVLFDRIRKGFPCEARVVARILPQFLDDFFPPQDIMNKVIGE    3000
Rattus          PSPATPDSESVIVAMERVSVLFDRIRKGFPCEARVVARILPQFLDDFFPPQDVMNKVIGE    2978
Mus             PSPATPDSESVIVAMERVSVLFDRIRKGFPCEARVVARILPQFLDDFFPPQDVMNKVIGE    2977
                      * * :    ::*.:*:. *:..    * : .::* *:: *:.* ::::.*** *

Drosophila      FLAINHPHCDVIAMIVYQVFRSAIDSSYLQMLQDWLICTLPTFLD-QPEQQGVWGLSVIF    3487
Takifugu        FLSNQQPYPQFMATVVYKVFQTLHATGQSSMVRDWVLLSLSNFTQRTPVAMAMWSLSCFF    3066
Danio           FLSNQQPYPQFMATVVYKVFQTLHATGQSSMVRDWVLLSLSNFTQRTPVAMAMWSLSCFF    3039
Bos             FLSSQQPYPQFMATVVYQVFQTLHGAGQSPMVRDWVMLSLSNFTQRSPVAMAMWSLSCFF    3052
Homo            FLSNQQPYPQFMATVVYKVFQTLHSTGQSSMVRDWVMLSLSNFTQRAPVAMATWSLSCFF    3060
Rattus          FLSNQQPYPQFMATVVYKVFQTLHSAGQSSMVRDWVMLSLSNFTQRTPVAMAMWSLSCFL    3038
Mus             FLSNQQPYPQFMATVVYKVFQTLHSAGQSSMVRDWVMLSLSNFTQRTPVAMAMWSLSCFL    3037
                **: ::*: :.:* :**:**::   :.   *::**:: :* .* :  *   . *.** ::

Drosophila      LSASINLHLIKLFPLVLGIGASNSAAAATTATTATTEAEAAAPAMARKLGQHEIALFVTA    3547
Takifugu        VSASTSQWISALLPHVISRM--------------------------GSSDVVDVNLFCLV    3100
Danio           VSASTSQWISALLPHVISRM--------------------------GKSDTVDISLFCLV    3073
Bos             VSASTSPWVSAILPHVVSRM--------------------------GKLEQVDVNLFCLV    3086
Homo            VSASTSPWVAAILPHVISRM--------------------------GKLEQVDVNLFCLV    3094
Rattus          VSASTSPWVSAILPHVISRM--------------------------GKLEQVDVNLFCLV    3072
Mus             VSASTSPWVSAILPHVISRM--------------------------GKLEQVDVNLFCLV    3071
                :*** .  :  ::* *:.                             .    :: **  .

Drosophila      AQDFHAKLSGEQ--RQRFREAFGSF-KRSQVYGRMLQCL---------3583
Takifugu        AMDFYRHQIDEELDRRAFQSVFETVASPGSPYFQLLACLQSIHQDKSL3148
Danio           AMDFYRHQIDEELDRRAFQSVFEMVASPGSPYYQLLCCLQSIHQDTSL3121
Bos             ATDFYRHQIEEELDRRAFQSVFEVVAVPGNPYHRLLACLRSVQKAAAC3134
Homo            ATDFYRHQIEEELDRRAFQSVLEVVAAPGSPYHRLLTCLRNVHKVTTC3142
Rattus          ATDFYRHQIEEEFDRRAFQSVFEVVAAPGSPYHRLLACLQNVHKVTAC3120
Mus             ATDFYRHQIEEEFDRRAFQSVFEVVAAPGSPYHRLLACLQNVHKVTTC3119
                * **: :   *:  *: *:..:  .   .. * ::* **
```

PLEASE NOTE: Showing colors on large alignments is slow.
